# *Phytophthora*, *Nothophytophthora* and *Halophytophthora* diversity in rivers, streams and riparian alder ecosystems of Central Europe

**DOI:** 10.1007/s11557-023-01898-1

**Published:** 2023-06-13

**Authors:** Tamara Corcobado, Thomas L. Cech, Andreas Daxer, Henrieta Ďatková, Josef Janoušek, Sneha Patra, Daniella Jahn, Christine Hüttler, Ivan Milenković, Michal Tomšovský, Marília Horta Jung, Thomas Jung

**Affiliations:** 1 Phytophthora Research Centre, Faculty of Forestry and Wood Technology, Mendel University in Brno, Zemědělská 3, 61300 Brno, Czech Republic; 2 Natural Hazards and Landscape, Unit of Phytopathology, Department of Forest Protection, Federal Research and Training Centre for Forests, Seckendorff-Gudent-Weg 8, 1131 Vienna, Austria; 3 Global Change Research Institute of the Czech Academy of Sciences, Belidla 986/4a, 603 00 Brno, Czech Republic; 4 Faculty of Forestry, University of Belgrade, Kneza Višeslava 1, 11030 Belgrade, Serbia; 5 Phytophthora Research and Consultancy, Am Rain 9, 83131 Nußdorf, Germany

**Keywords:** Oomycetes, Hybrids, Alpine, Dieback, Water filtration, *Phytophthora plurivora*

## Abstract

Waterways are ideal pathways for *Phytophthora* dispersal and potential introduction to terrestrial ecosystems. While many *Phytophthora* species from phylogenetic clades 6, 9 and 10 are predominant oomycetes in watercourses due to their adaptation to a lifestyle as saprotrophs and opportunistic pathogens of riparian plants, species from clades 2, 7 and 8 are predominantly soil- or airborne using aquatic habitats as temporal niches for spreading and invading terrestrial sites along the watercourses. In contrast to forest ecosystems, knowledge of *Phytophthora* diversity in watercourses in Central Europe is limited. Between 2014 and 2019 extensive surveys of streams and rivers were undertaken across Austria, in South Moravia, Czech Republic and Žilina province, Slovakia to unveil the diversity and distribution of *Phytophthora* and related oomycetes. In addition, in Austria riparian forests of black alder (*Alnus glutinosa*) and grey alder (*A. incana*) in lowlands and in the Alps were examined. A variety of *Phytophthora* species from clades 2, 6, 7, 8, 9 and 10 were isolated, with clade 6 species showing the widest distribution and abundance. Furthermore, interspecific clade 6 hybrids and other oomycetes such as *Halophytophthora fluviatilis* and undescribed *Nothophytophthora* spp. were also obtained. In riparian alders, symptoms of *Phytophthora* infections were associated with species from the *P.* × *alni* complex and *P. plurivora*. *Phytophthora plurivora* was most common in alder stands whereas *P. uniformis* was the oomycete species occurring at the highest altitude in alpine riparian areas.

**Supplementary Information:**

The online version contains supplementary material available at 10.1007/s11557-023-01898-1.

## Introduction

Many species from the oomycete genus *Phytophthora* are responsible for the destruction of natural and semi-natural forests and woodlands at a global scale (Jung et al. 2018a; Brasier et al. 2022). Until the 1990s, *Phytophthora* species were mainly reported as pathogens of orchards and horticultural crops (Kreutzer et al. 1940; Deutschmann 1954; Hildebrand 1959; Tsao 1990; Bourke 1991) and infrequently as pathogens affecting forest ecosystems (Tucker and Milbrath 1942; Podger et al. 1965). However, since the 1960s the number of important diseases of forests and other natural ecosystems caused by invasive *Phytophthora* species has increased exponentially from six to currently 41 (Brasier et al. 2022). Since water enhances the pathogen's sporulation, spread and infection via zoospores (Hirst and Stedman 1960; McIntosh 1964; Lacey 1967; Chen et al. 2022) isolations of *Phytophthora* from irrigation water in crops and nurseries were performed for monitoring and clarifying the diversity of *Phytophthora* pathogens (Klotz et al. 1959; Thomson and Allen 1974; Oudemans 1999; Bush et al. 2003). The use of surface water for irrigation can introduce *Phytophthora* spp. to nurseries and horticultural areas and facilitate the infection of plants while recirculation systems can lead to an accumulation and concentration of *Phytophthora* inoculum enabling spread of *Phytophthora* pathogens from infested to non-infested plants, thus exacerbating the pathogen's detrimental effects. Early studies on *Phytophthora* presence in surface water of forests were performed by Kliejunas and Ko (1976) and von Broembsen (1984). These showed the occurrence of *P. cinnamomi* in run-off water and standing water (i.e. puddles) and concluded that the water would act as vehicle for pathogen spread to new locations posing a potential risk for nurseries and agricultural systems. In the early 2000s, an extensive stream monitoring program has been initiated in the USA to promote early detection of *P. ramorum*, the causal agent of the devastating sudden oak death (Sutton et al. 2009; Reeser et al. 2011) and to investigate the ecology and pathways of this pathogen (Murphy et al. 2008; Hwang et al. 2008, 2011). Subsequently, also in other continents studies of *Phytophthora* diversity in watercourses were carried out (e.g. Hüberli et al. 2013; Oh et al. 2013; Matsiakh et al. 2016). First records of *Phytophthora* pathogens causing devastating disease outbreaks and mortality along rivers in European forests was associated with alder trees (*Alnus* spp.) in the mid-1990s (Brasier et al. 1995; Gibbs et al. 1999; Hartmann 1995; Streito et al. 2002; Jung and Blaschke 2004). In Europe, the first stream surveys complemented with forest surveys were performed by Hansen and Delatour (1999) in France. Simultaneous *Phytophthora* surveys in aquatic and terrestrial ecosystems have subsequently been carried out in Sicily, Sardinia, Chile, South Africa, Taiwan and Vietnam (Nagel et al. 2013, 2015; Oh et al. 2013; Jung et al. 2017a, b, c, 2018b, 2019, 2020; Bregant et al. 2020).

Among riparian trees along riversides, only a few tree species are known to be susceptible to *Phytophthora*. In natural ecosystems, *Fraxinus* spp. was recorded to suffer from dieback and mortality associated with *Phytophthora* infections (Orlikowski et al. 2011; Akilli et al. 2013). Undoubtedly, tree species from the genus *Alnus* are recognized as the most vulnerable to *Phytophthora* attacks on riparian sites. They are affected by several species of *Phytophthora*, including *P. plurivora*, *P. gonapodyides*, *P. polonica*, *P. lacustris*, *P. syringae*, *P. pseudosyringae*, *P. cactorum*, *P. siskiyouensis*, *P. hydropathica*, *P. bilorbang* and especially the *P.* × *alni* species complex, i.e. *P. uniformis*, *P.* × *alni* and *P.* × *multiformis*, which show host-specificity to alders (Brasier and Kirk 2001; Jung et al. 2003; Jung and Blaschke 2004; Brasier et al. 2004; Štěpánková et al. 2013; Perez-Sierra et al. 2015; Trzewik et al. 2015; Matsiakh et al. 2021). More recently, *P. acerina*, *P. pseudocryptogea*, *P.* × *serendipita* and the newly described *P. alpina* were reported causing disease symptoms on *Alnus* spp. (Bregant et al. 2020; Seddaiu and Linaldeddu 2020). In Europe, alder decline has been recorded and studied mainly in black alder (*A. glutinosa*) and to a much lesser extent in grey alder (*A. incana*) (Gibbs et al. 1999; Streito et al. 2002; Jung and Blaschke 2004; Černý and Strnadová 2010; Solla et al. 2010; Trzewik et al. 2015; Seddaiu and Linaldeddu 2020). However, other alder species, such as *A. viridis* and *A. cordata* are also susceptible to *Phytophthora* (Santini et al. 2001; Jung and Blaschke 2006; Bregant et al. 2020). Grey alder commonly occurs in Northern and Central Europe and is particularly widespread in alpine areas of intermediate to high altitude (Houston Durrant et al. 2016a). Black alder is widespread in lowland areas across Europe but can also grow at higher altitudes (Houston Durrant et al. 2016b). Since it is currently little known about the extent of *Phytophthora*-mediated decline of both alder species in alpine areas (Bregant et al. 2020) it is of interest to investigate the severity of alder decline in countries which include alpine regions with a high frequency of riparian alders.

To date, knowledge of *Phytophthora* diversity and distribution within Central European waterways is still very limited. Also, little is known about *Phytophthora* distribution in riparian alder ecosystems in Austria (Cech 2006; Balci and Cech 2004). Using various isolation methods, this study aimed to unveil the *Phytophthora* species assemblage in (i) rivers, streams and associated alder forests in Austria at different time points; and (ii) forest streams of the southern region of the Czech Republic (South Moravia) and the north-western region of Slovakia (Žilina).

## Materials and methods

### Sampling sites

**Fig. 1:**
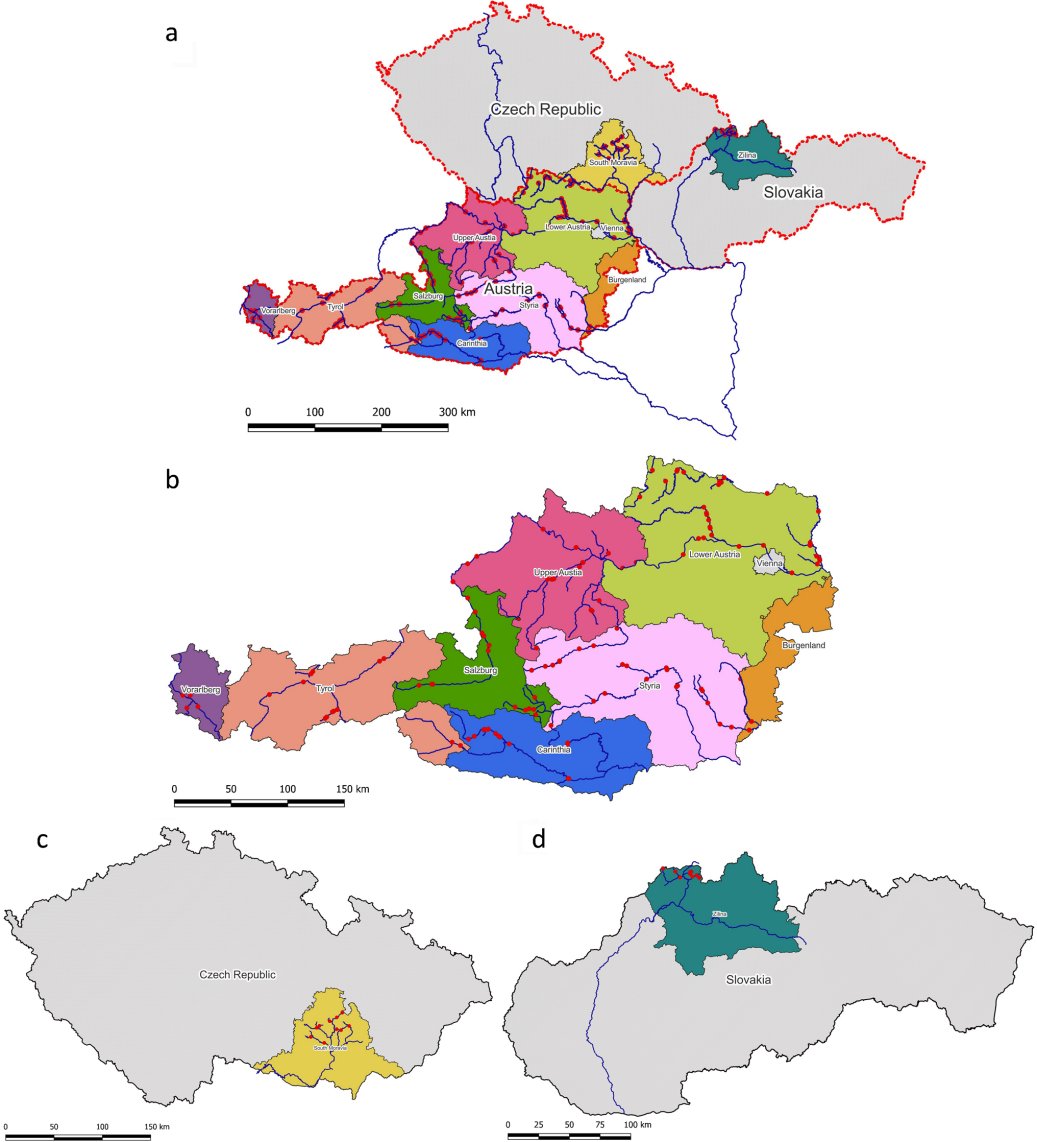
Distribution of sites (**a**) included in the oomycete surveys of watercourses and riparian alder forests in Austria (**b**), Czech Republic (**c**) and Slovakia (**d**)

The surveys and samplings were accomplished in watercourses and in the riparian alder forests along the rivers and streams in Austria during spring and summer 2014, summer 2015, summer and autumn 2016 and autumn 2019; and in rivers and streams of South Moravia, Czech Republic, and Žilina, Slovakia, during November 2018 (Table S1 and S2, Fig. 1). Sampling along watercourses was performed at sites with easy access to the riparian forests and the banks (Table S1). In situ stream baitings were carried out in locations with reduced visibility (e.g. under bridges or overhanging branches) and where water flow was calm to facilitate zoospore attachment to baiting leaves.

**Fig. 2:**
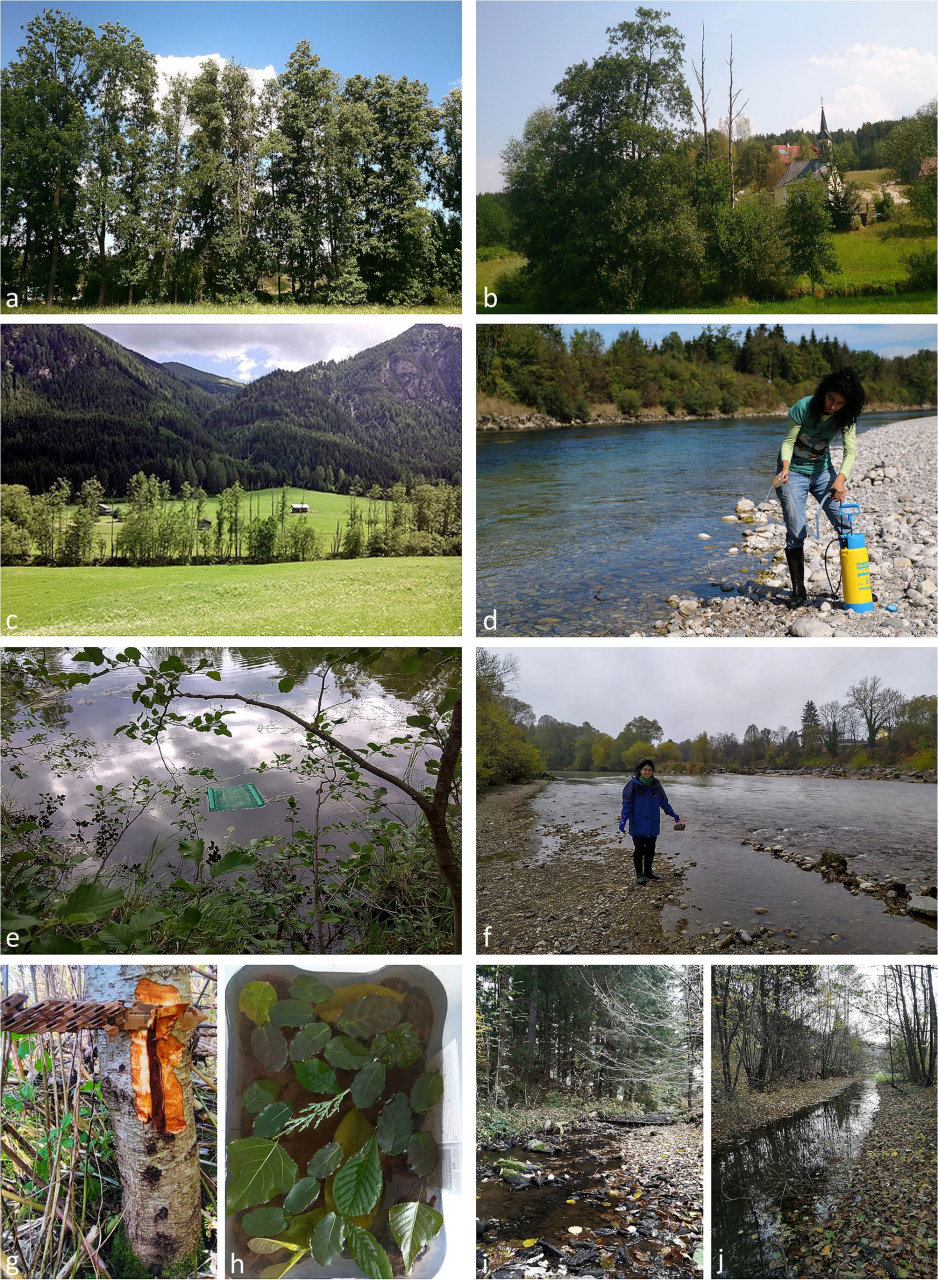
Oomycete survey of riparian black and grey alder sites and waterways in Austria, the Czech Republic and Slovakia, with emphasis on *Phytophthora* spp.; (**a**) crown defoliation of *Phytophthora*-infected black alders in Austria; (**b**) crown dieback of *Phytophthora*-infected black alder; (**c**) *Phytophthora*-infected grey alders along an alpine stream showing different levels of decline; (**d**) water filtration in Austria; (**e**) baiting raft floating in an Austrian stream; (**f**) collection of floating naturally fallen leaves at an Austrian river; (**g**) bleeding stem canker on a young black alder in Austria; (**h**) soil baiting using leaves from several tree species as baits; (**i**) floating naturally fallen leaves in a stream of the Czech Republic; (**j**) floating naturally fallen leaves in a Slovakian stream

In Austria, we selected 37 rivers and streams distributed across all nine states along a wide altitude gradient from lowland to alpine areas (140.0–1408.7 m a.s.l.) (Figs. S1-S8). Four rivers and one stream are eventually draining into the North Sea (i.e. Lainsitz, Ill, Alvier, Lutz and Reißbach) while the remaining rivers and streams are draining into the Black Sea. In addition, 57 riparian forest sites with the presence of black or grey alder trees were sampled. Sampling was performed by selecting 20–100 m long sections along the riverbanks containing black or grey alder trees with typical symptoms of *Phytophthora* infections such as crown defoliation and dieback, yellowing of leaves, small leaves, excessive fructification, bleeding stem cankers and rusty or tarry spots on the outer bark (Fig. 2). In 17 of the 57 riparian sites detailed assessments were performed. A total of 824 trees were examined thoroughly and classified into non-declining (less than 15% crown transparency and absence of bleeding stem cankers), declining (between 15 and 95% crown transparency and/or presence of bleeding stem cankers) and dead (more than 95% crown defoliation) trees. Other riparian tree species present at the sites included *Fraxinus excelsior*, *Populus* spp., *Salix* spp. and *Ulmus* spp., while more distant from the sites *Carpinus betulus*, *Quercus* spp., *Acer* spp., *Tilia* spp. and *Picea abies* occurred.

In both South Moravia and the Žilina regions each ten rivers and streams were selected (Table S1 and S2). The sites in South Moravia and Žilina were covering an altitudinal range of 242–466 m and 444–775 m a.s.l, respectively. All streams are draining into the Danube and eventually into the Black Sea (Figs. S9-S10).

### Sampling methods and *Phytophthora* isolation

In Austria six different sampling methods were used to provide a detailed overview of the *Phytophthora* diversity in rivers, streams and associated alder forests, i.e. in situ baiting, water filtration and collecting naturally fallen leaves, rhizosphere soil, fine roots and bark cankers.

For in situ baiting of rivers and streams, 35 × 40 cm baiting rafts, constructed from fly mesh and styrofoam, were filled each with 15–20 young leaves of the native species *Fagus sylvatica*, *Quercus robur*, *Acer* spp., *Alnus* spp., *Prunus avium* and *Aesculus hippocastanum*, tied with a rope to a shrub or tree at the riverbank and left for 2–3 days floating on the watercourses (Reeser et al. 2011; Jung et al. 2017a, b, c, 2018a, b, 2019). This method was used at 50 sites along 28 watercourses. In addition, naturally fallen necrotic leaves were collected from the water surface at 18 sites along 16 watercourses.

In South Moravia, Czech Republic, and the Žilina region of Slovakia each 30 naturally fallen necrotic leaves were collected from the water surface of the 20 forest streams. In South Moravia the leaves belonged to *Acer* spp., *Alnus* spp., *Carpinus betulus*, *Cornus* spp., *Corylus avellana*, *Fraxinus excelsior*, *Populus* spp., *Prunus* sp., *Quercus* spp., *Robinia pseudacacia*, *Salix* spp., *Tilia* spp. and *Ulmus* spp.. In Slovakia leaves of *Acer* spp., *Alnus* spp., *Corylus avellana*, *Fagus sylvatica*, *Populus* spp., *Prunus* sp., *Salix* spp. and *Picea abies* were sampled.

Collected baiting leaves and naturally fallen leaves were transported in cool boxes to the laboratory. Then the leaves were blotted dry, cut into small pieces and plated onto selective PARPNH agar (V8 juice agar (V8A) amended with 10 µg mL^−1^ pimaricin, 200 µg mL^−1^ ampicillin, 10 µg mL^−1^ rifampicin, 25 µg mL^−1^ pentachloronitrobenzene (PCNB), 50 µg mL^−1^ nystatin and 50 µg mL^−1^ hymexazol) (Jung et al. 2017a, b, c). The Petri dishes were kept at 20 ºC in the dark and checked for growing oomycete hyphae after 24–48 h. Oomycete hyphae were transferred from the growing margins onto V8A.

Water filtrations, according to Redondo et al. ([Bibr CR111][Bibr CR112]), were performed at 29 sites along 13 rivers in Austria. At each site, 10 L of water were collected and pumped with an agricultural 5 L volume hand sprayer through a 8 µm membrane (Merck Millipore, Cork, Ireland) inserted into a polysulfone filter holder (Whatman ™ Polysulfone Filter Holders, Syringe Type, Krackeler scientific, New York, USA). Several membranes were used to filter 10 L since particle accumulations obstructed the membranes. Between sampling sites, the pump was washed and disinfested by rinsing it several times with a mixture of 5% sodium hypochlorite and distilled water to avoid possible cross-contamination. The used membranes were stored in plastic bags and transported in a cool box to the laboratory where they were stored at 6 °C until processing on the following day. Membranes were blotted dry, cut into 1 × 1 cm pieces and plated onto selective PARPNH agar. The Petri dishes were kept at 20 ºC in the dark and oomycete hyphae were subcultured as described before.

In Austria, rhizosphere soil sampling and oomycete isolations were performed following the methodology of Jung (2009). In the selected sections of declining riparian alder forests, symptomatic and/or asymptomatic trees were sampled by collecting at each sample tree soil monoliths from 3–4 cardinal directions in 30–50 cm distance from the stem base and at a depth of 10–30 cm avoiding the superficial organic layer, and bulking them to a composite sample of ca 1–2 L. In total 64 composite rhizosphere soil samples were transported to the lab. Well-mixed subsamples of circa 300 mL per composite soil sample were transferred to plastic containers and flooded with distilled water so that the water surface was approximately 3 cm above the soil surface. After cleaning the water surface from floating particles with kitchen paper tissue, young leaflets of the same native tree species used in the baiting rafts were placed as baits to float on the water surface. Infected necrotic leaflets were then blotted dry, cut into small fragments and plated onto selective PARPNH agar. Petri dishes were kept at 20 ºC in the dark and oomycete hyphae were subcultured as described before.

Active bleeding bark lesions and, infrequently, also fine roots of declining alder trees were sampled according to Jung and Blaschke (2004) and Jung (2009). The bark samples, including the cambium, were taken from the upper section of fresh lesions using a hammer and a chisel and transported in wide-mouth bottles with distilled water to the lab. The water was exchanged 3–4 times per day over two days to remove excess polyphenols. Then small pieces of bark were cut from active parts of the lesions, blotted dry and plated onto selective PARPNH agar. Furthermore, fine roots from rhizosphere soil samples of 2–3 trees were collected at five declining alder sites. Roots were cleaned under running water, blotted on filter paper and small sections plated onto selective PARPNH agar. Petri dishes were kept at 20 ºC in the dark and oomycete hyphae were subcultured as described before.

Species identification of oomycete isolates was performed using molecular sequence analyses (see below). For isolates identified as *P.* × *alni* / *P.* × *multiformis*, a morphological characterisation of 3 weeks old V8A cultures at × 320 under the light microscope was performed to distinguish between *P.* × *alni* and *P.* × *multiformis* (Brasier et al. 2004).

### Molecular identification

DNA extraction was performed using a magnetic bead-based technology. The protocol “BOMB TNA extraction from plants using TNES/GITC lysis” (high throughput protocol; Oberacker et al. 2019) was modified to extract DNA from the isolates (Jung et al. 2022). Mycelium from a colony growing on nutrient media for one up to three weeks was collected and transferred to 2 mL homogenisation tubes (116910500, MP Biomedicals; Irvine, USA) which were stored at -80 °C until further use. Homogenisation was performed at 5 800 RPM 4 × 15 s using Precellys Evolution instrument (Bertin Technologies; Montigny-le-Bretonneux, France). The samples were centrifuged briefly to collect the content at the bottom and 60 µl of TNES buffer with RNase A (T3018L; New England Biolabs; Ipswich, USA) was added. The mixture was incubated for 20 min at 56 °C. 240 µl of 1.5 × GITC lysis buffer was added and mixed thoroughly. The mixture was centrifuged for 10 min at 10 000 RPM and 180 µl of supernatant was transferred to a deep-well block (82.1970.002; Sarstedt; Nümbrecht, Germany). DNA was further mixed with 120 µl of AMPure XP beads (A63881; Beckman Coulter; Indianapolis, USA) and 240 µl of isopropanol and incubated for 10 min. All washing steps were performed according to the original protocol. DNA was eluted in 70 µl of TE buffer (12090015; Invitrogen; Carlsbad, USA) and stored at -80 °C for long-term preservation.

Initial identification was performed with ITS gene region sequence analysis using ITS1/4 (White et al. 1990) or ITS6/4 (Cooke et al. 2000) primers. The *cox1* and ß-tubulin genes were sequenced using OomCoxI-Levup/OomCoxI-Levlo (Robideau et al. (2011) and TUBUF2/TUBUR1 (Kroon et al. 2004), respectively. PCR conditions and gel electrophoresis were as described by Jung et al. (2022). PCR products were purified and sequenced by Eurofins Genomics (Ebersberg, Germany) in both directions with the primers used for PCR amplification. DNA sequence data from representative isolates from this study were deposited in GenBank and their accession numbers are given in Table S3.

## Results

### *Phytophthora* diversity and distribution in rivers and streams of Austria, South Moravia (Czech Republic) and Žilina (Slovakia)

**Fig. 3:**
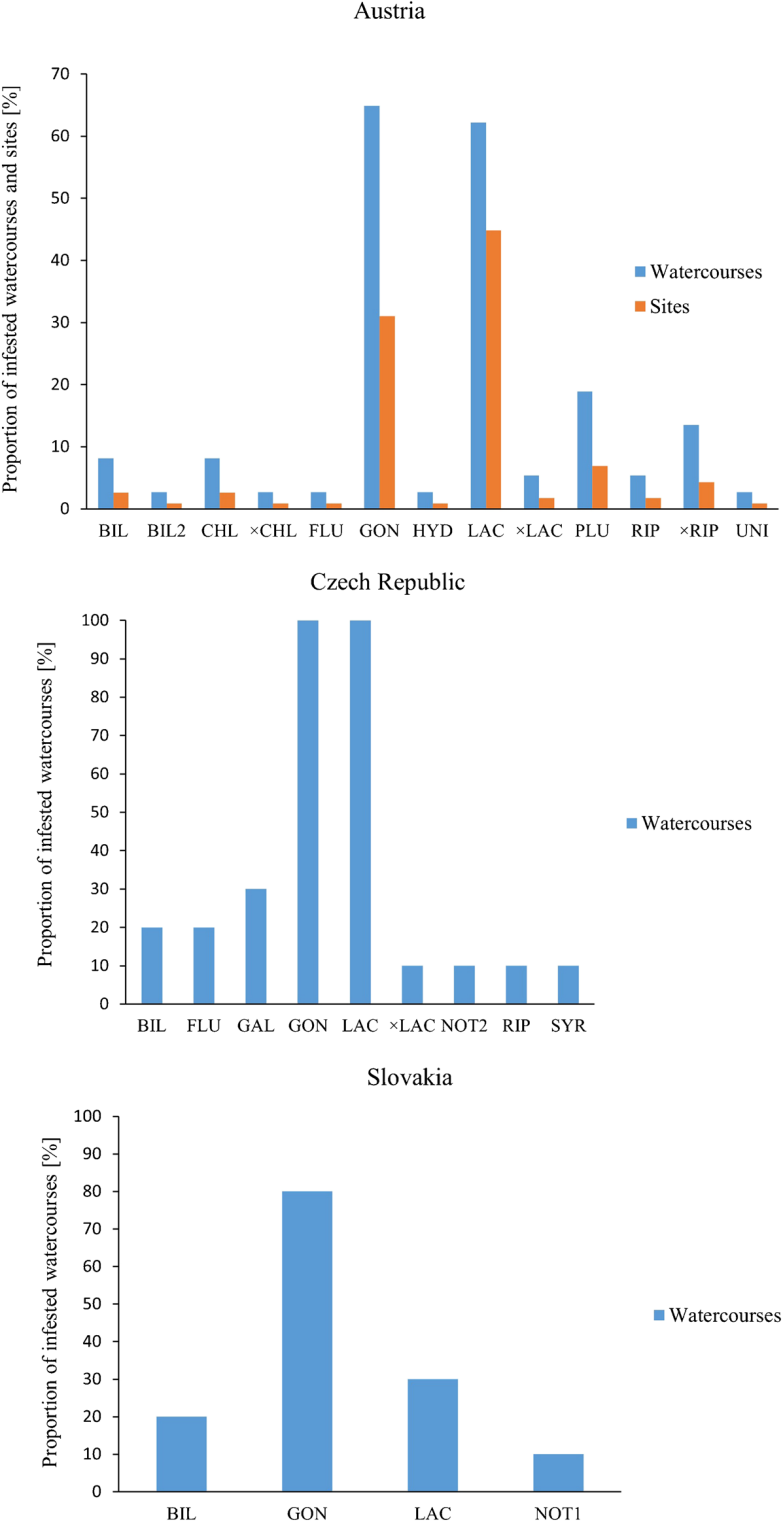
Aquatic diversity of *Phytophthora*, *Halophytophthora* and *Nothophytophthora* species in watercourses and riparian sites in Austria, Czech Republic and Slovakia; BIL = *P. bilorbang*, BIL2 = *P.* taxon *bilorbang*-like 2, CHL = *P. chlamydospora*, × CHL = *P. chlamydospora* × *lacustris*, FLU = *Halophytophthora fluviatilis*; GAL = *P. gallica*; GON = *P. gonapodyides*, HYD = *P. hydropathica*, LAC = *P. lacustris*, × LAC = *P.* taxon × *lacustris*; NOT1 = *Nothophytophthora* taxon 1; NOT2 = *Nothophytophthora* taxon 2; PLU = *P. plurivora*, RIP = *P. riparia*, × RIP = *P.* taxon × *riparia*, SYR = *P. syringae*, UNI = *P. uniformis*

In Austria, 10 *Phytophthora* taxa and three *Phytophthora* hybrid taxa belonging to phylogenetic clades 2c, 6b, 7a and 9a, as well as *Phytopythium litorale* and a *Halophytophthora* species were obtained from 29 of 37 rivers and streams (78.4%) corresponding to 45 of 95 sites (47.4%) (Table S1; Fig. 3). In addition, several *Pythiaceae* species were isolated (e.g. *Elongisporangium undulatum*). Using baiting rafts, from 22 of the 28 rivers (78.6%) and at 32 of the 50 sites (64%) where this method was used, seven *Phytophthora* species including *P. chlamydospora*, *P. gonapodyides*, *P. hydropathica*, *P. lacustris*, *P. plurivora*, *P. riparia* and *P. uniformis* and two hybrid taxa, i.e. *P.* taxon × *riparia* and *P. chlamydospora* × *lacustris*, were isolated. From naturally fallen floating leaves four *Phytophthora* taxa, two hybrids and one *Halophytophthora* species were isolated from 14 of the 16 rivers (87.5%) and at 15 of the 18 sites (83.3%) surveyed by this method. The species assemblage included *P. bilorbang*, *P. gonapodyides*, *P. lacustris*, the new yet undescribed *P.* taxon bilorbang-like 2, *P.* taxon × *lacustris*, *P.* taxon × *riparia* and *Halophytophthora fluviatilis*. Using the water filtration method, *P. chlamydospora, P. gonapodyides*, *P. lacustris*, *P. plurivora*, *P. riparia* and *P.* taxon × *riparia* were obtained from 9 of 13 rivers (69.2%) and at 19 out of 29 sites (65.5%) where this isolation method was applied.

*Phytophthora* species from phylogenetic clade 6b with a primarily aquatic lifestyle showed the widest distribution in aquatic ecosystems across Austria. Using the three sampling methods they were found in 78.4% of the 37 rivers and streams. Overall, the clade 6b species *P. gonapodyides* (64.9% of all waterways) and *P. lacustris* (62.2%) were the most widespread species followed by *P. plurivora* from clade 2c (18.9%) and the clade 6b hybrid *P.* taxon × *riparia* (13.5%). All other *Phytophthora* species had a scattered distribution.

**Fig. 4:**
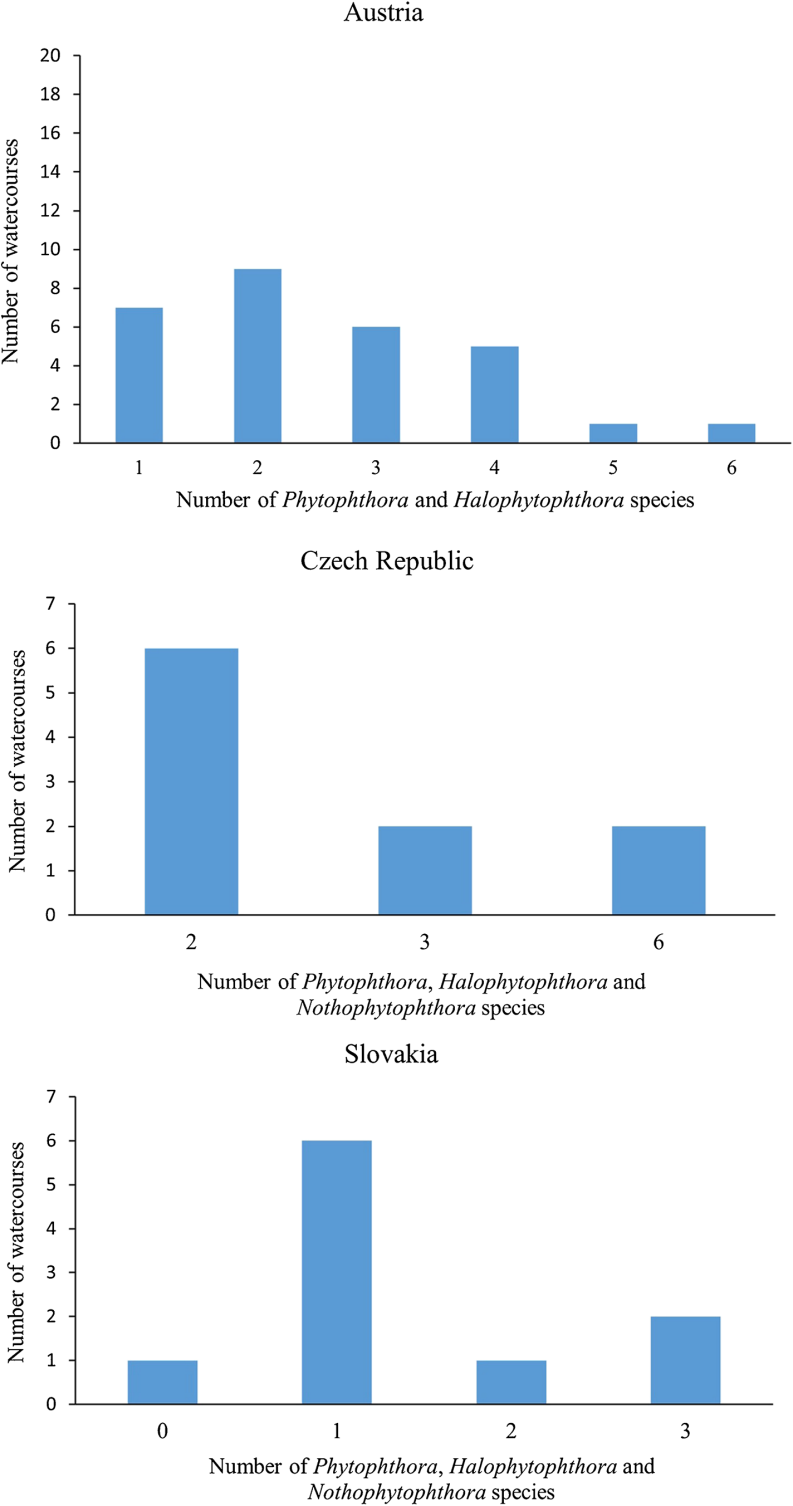
Numbers of watercourses in Austria, Czech Republic and Slovakia with different total numbers of oomycete species

The Austrian watercourses with the highest numbers of *Phytophthora* taxa (Fig. 4) were the Kamp (6 taxa = 46.2% of the total number of taxa) and the Krumme Steyerling (5 taxa; 38.5%) followed equally by Traun, Enns, Zeller Ache (Ager) and Stempfelbach (4 taxa; 30.8%).

*Phytophthora gonapodyides* occurred between 141.1 and 1292.8 m a.s.l. and, hence, was the species with the widest altitudinal amplitude and the highest elevation, specifically in Thomatalerbach (1292.8 m), Leutascher Ache (1096.7 m) and Mur (1137.0 m). *Phytophthora lacustris* (140.0–690.8 m), *P.* taxon × *lacustris* (141.1–348.1 m) and *P. plurivora* (240.9–690.8 m) had a narrower altitudinal amplitude. The new undescribed *P.* taxon bilorbang-like 2 was recovered at an altitude of 140.4 m.

In South Moravia, all 10 sampled streams (10 sites) were positive for *Phytophthora* using naturally fallen leaves as baits. In total six *Phytophthora* taxa from clades 6b, 8d and 10 and one hybrid taxon from clade 6b were found (Table S1; Fig. 3). *Phytophthora gonapodyides* and *P. lacustris* were present in all watercourses followed by the clade 10 species *P. gallica* (3 streams) and *P. bilorbang* from clade 6b (2 streams) while *P. syringae* from clade 8d and the clade 6b taxa *P. riparia* and *P. lacustris* × *riparia* occurred only in one stream each. In addition, a new yet undescribed *Nothophytophthora* species, informally designated as *Nothophytophthora* taxon 2, and *H. fluviatilis* were obtained from one and two streams, respectively. *Phytopythium citrinum*, *Phy. litorale* and other undescribed *Phytopythium* taxa were also present in these streams. The streams with the highest number of *Phytophthora* taxa (Fig. 4) were the Kuřimka (6 taxa = 85.7% of the total number of species) and the Punkva (5 taxa; 71.4%) followed by Časnýř and Říčka streams (3 taxa; 42.9%). The ten streams were within a narrow altitudinal range of 242–466 m.

In the Žilina region of Slovakia, three *Phytophthora* species from clade 6b were isolated from 8 of the 10 surveyed streams/sites (Table S1; Fig. 3). In one of the streams where *Phytophthora* was not found, the new yet undescribed *Nothophytophthora* taxon 1 was obtained. *Phytophthora gonapodyides* occurred in all eight *Phytophthora*-positive streams (80% of all streams) while in the western and north-western parts of Žilina *P. lacustris* and *P. bilorbang* were present in two and three streams, respectively. Other oomycete taxa included *Phy. litorale*, *Pythium kashmirense* and other undescribed *Phytopythium* and *Pythium* spp. The highest numbers of *Phytophthora* taxa (Fig. 4) were found in the streams Črchľový potok and in Predmieranka (3 taxa = 100% of the total number of taxa). From Predmieranka stream with the highest altitude (775.0 m) of all streams sampled in Slovakia, *P. gonapodyides*, *P. lacustris* and *P. bilorbang* were isolated.

### *Phytophthora* diversity and distribution in riparian alder forests in Austria and association with disease symptoms

Alder trees showed decline symptoms in most of the selected 57 riparian alder forest sites. Across the 17 sites which were studied in detail, on average 36.0% of the 824 assessed *Alnus* trees were showing crown defoliation and/or stem cankers, 11.6% were dead and 52.4% were healthy. Overall, 9 *Phytophthora* taxa belonging to phylogenetic clades 1a, 2c, 6b, 7a, 8d and 9a were present in rhizosphere soil or bark cankers at 26 of the 57 riparian black alder and grey alder forest sites sampled in Austria (Table S1). Six *Phytophthora* species were isolated from rhizosphere soil samples of 26 of the 64 trees sampled (40.6%) including *P. cactorum*, *P. gonapodyides*, *P. lacustris*, *P. plurivora*, *P. syringae* and *P. uniformis*. Two *Phytophthora* species, i.e. *P. lacustris* and the clade 9a species *P. polonica*, were isolated from fine roots of 3 of the 25 trees sampled (12%). Three alder-specific *Phytophthora* species from clade 7a were isolated from bark lesions of 17 of the 38 trees sampled (44.7%), including *P.* × *alni* (5 sites, 7 trees), *P.* × *multiformis* (2 sites, 2 trees) and *P. uniformis* (5 sites, 5 trees).

**Fig. 5:**
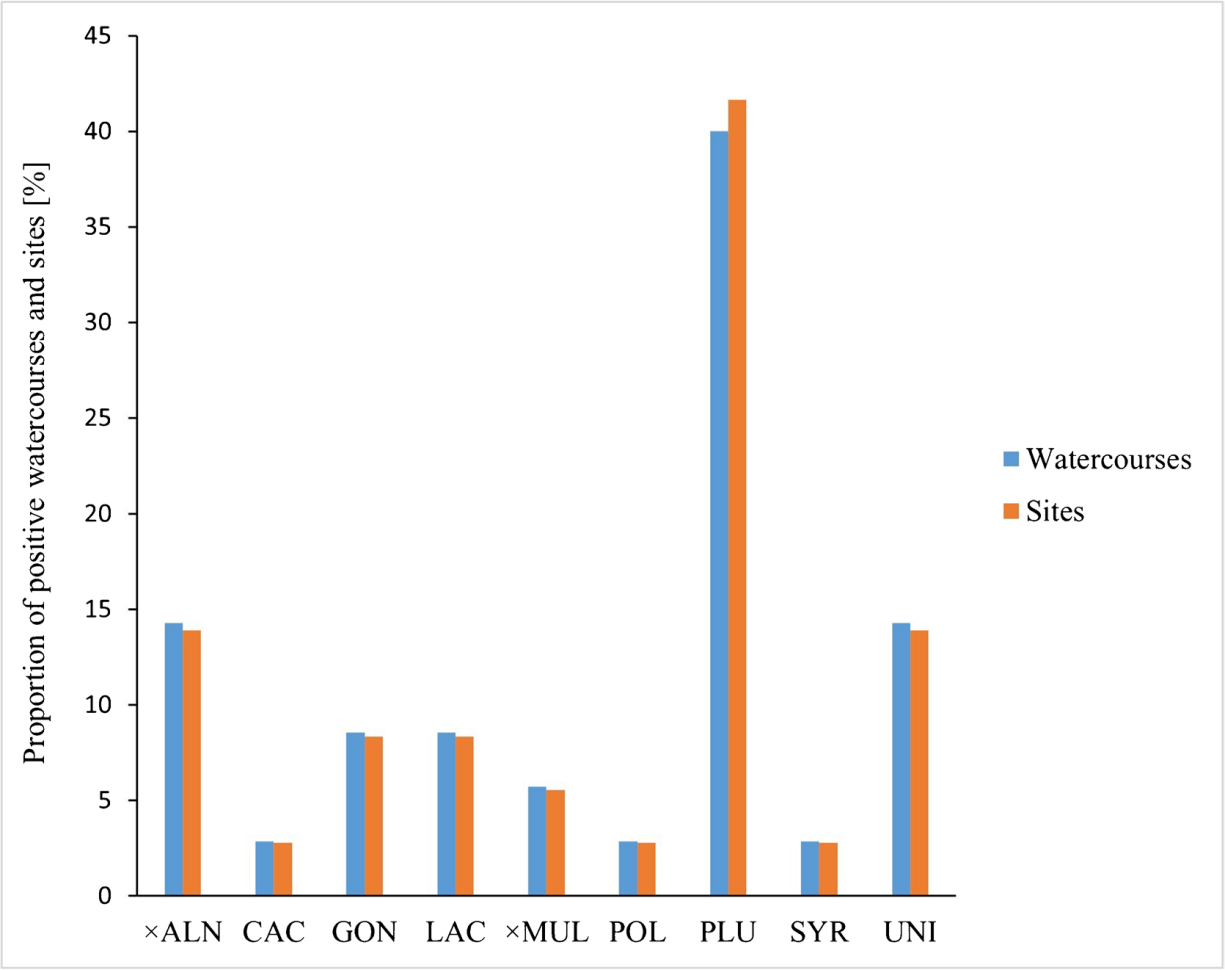
Diversity and frequency of *Phytophthora* species isolated from rhizosphere soil and bark cankers of alder trees in riparian forests in Austria; × ALN = *P.* × *alni*, CAC = *P. cactorum*, GON = *P. gonapodyides*, LAC = *P. lacustris*, × MUL = *P.* × *multiformis*, PLU = *P. plurivora*, POL = *P. polonica*, SYR = *P. syringae*, UNI = *P. uniformis*

The 57 riparian forest sites were associated with 30 rivers and streams. With 15 alder forest sites along 14 rivers/streams *P. plurivora* showed the widest distribution of all species (Fig. 5). *Phytophthora plurivora* was recovered from rhizosphere soil samples but not from bark cankers. With nine riparian forests associated with nine rivers/streams *P.* × *alni*, *P.* × *multiformis* and *P. uniformis* were also widespread. Except for *P. uniformis*, which was also obtained from a rhizosphere soil sample, they were exclusively isolated from alder bark cankers. *Phytophthora* × *alni* and *P. uniformis* occurred each in five riparian forest sites along five rivers/streams, co-occurring at the river Inn (different sites) and the stream Marbach (same site). *Phytophthora* × *multiformis* was found in two forest sites at two rivers. *Phytophthora gonapodyides* and *P. lacustris* were each recovered from three riparian forest sites at three rivers, co-occurring at the river Drau. While *P. gonapodyides* was only isolated from rhizosphere soil, *P. lacustris* was obtained from both fine roots and rhizosphere soil. *Phytophthora* species from phylogenetic clades 1a (*P. cactorum*), 8d (*P. syringae*) and 9a (*P. polonica*) occurred only in each one riparian forest site.

The highest numbers of *Phytophthora* species were found in the alder forest sites associated with the river Inn (4 species = 44.4% of the total number of species) followed by the rivers Enns and Salzach (3 species; 33.3%), and the Danube, Drau, Lungitzer Loben, Marbach, Reißbach and Weizbach (2 species; 22.2%) (Table S1).

The most widespread and common species, *P. plurivora*, occurred at forest sites with an altitudinal range of 144.5–793.7 m. *Phytophthora uniformis* was the species with the widest altitudinal amplitude (311.8–1266.3 m) occurring even at the highest forest sites along the streams Turrach (1266.3 m) and Gschnitzbach (1081.9 m). The other two clade 7a species, *P.* × *alni* (311.8–782.4 m) and *P.* × *multiformis* (149.3–504.3 m), were restricted to considerably lower altitudes. Interestingly, although *P. gonapodyides* showed the widest altitudinal range and the highest altitude in the stream surveys, in alder forest sites it was only found between 495.8 and 730.9 m a.s.l.

Generally, *Phytophthora* was isolated from declining alder trees where typical symptoms of infection were noted. The most affected states with a substantial number of observations of declining alders were Styria, Tyrol and Salzburg. Lower Austria, Upper Austria and Burgenland showed an intermediate incidence of disease while only healthy alder stands were recorded in the state of Voralberg. The most common and widespread *Phytophthora* species in the riparian alder forests was *P. plurivora*; it is notable that its isolation was restricted to rhizosphere soil samples whereas active bleeding cankers did not yield *P. plurivora*. Nonetheless, in most cases, an intermediate to a high incidence of alder decline was associated with its presence (e.g. sites 100 and 109 with 20 and 100% declining or dead alder trees, respectively). At grey alder site 50 on the banks of the river Inn (Tyrol), where *P. plurivora* was isolated from the rhizosphere soil of a declining tree, 140 out of 200 trees were declining with 20% of them showing old inactive stem cankers with tarry spots. *Phytophthora* × *multiformis* was exclusively isolated from two sites with high incidences of alder mortality (i.e. sites 4 and 128 with 18 and 52% dead alder trees, respectively), co-occurring with *P.* × *alni* at one of the sites. Sites, where *P.* × *alni* (e.g. sites 131 and 50) or *P. uniformis* (e.g. site 87) occurred, were usually linked with intermediate to high levels of alder decline. Co-occurrence of *P.* × *alni* and *P. uniformis* caused particularly high mortality (e.g. site 129 with 58% mortality).

## Discussion

This study provides for the first-time insights into the *Phytophthora* diversity of watercourses in Central Europe, namely in Austria and in each one region of Czech Republic (South Moravia) and Slovakia (Žilina), respectively. It also shows the first extensive overview of *Phytophthora* occurrence in riparian black and grey alder forests across Austria. *Phytophthora* diversity was higher in the 95 watercourses as compared to the 57 terrestrial alder ecosystems surveyed in Austria. This is a common pattern in combined aquatic and terrestrial *Phytophthora* surveys (Oh et al. 2013; Jung et al. 2017a, 2018b, 2019, 2020; Seddaiu et al. 2020) as watercourses provide ideal niches for aquatic *Phytophthora* species and also temporal niches for various soil- and airborne *Phytophthora* species. Aquatic *Phytophthora* species are mainly saprotrophs on floating and submerged organic debris which sporadically can establish in terrestrial ecosystems as comparatively weak opportunistic tree pathogens. Soilborne *Phytophthora* species spend most of their life cycle as root and bark pathogens of terrestrial plant species but most of them can also survive in watercourses as saprotrophs and spread downstream to other terrestrial ecosystems. Among those *Phytophthora* species found in the watercourses in Austria, Czechia and Slovakia, typical aquatic clade 6 species were most common and abundant. Aquatic clade 6 species are able to colonize green leaves and dead or decomposing leaf litter (Brasier et al. 2003; Jung et al. 2011) and due to their high competitiveness overtake the colonization of other species (Aram and Rizzo 2018). Other aquatic species were *P. hydropathica* (clade 9) and *P. gallica* (clade 10) which occurred in Austria and South Moravia, respectively. The other *Phytophthora* species recovered from watercourses in Central Europe belong to clades 2, 7, 8 and 9 and are known to have a predominantly soilborne lifestyle. *Phytophthora plurivora* from clade 2 occurred in seven Austrian rivers/streams and has previously been recorded from rivers and streams in Chile, Ireland, Italy and the USA (Reeser et al. 2011; O' Hanlon et al. 2016; Jung et al. 2018b, 2019; Seddaiu et al. 2020; Riolo et al. 2020), demonstrating its ability to survive and spread in aquatic ecosystems. Its widespread occurrence in watercourses is concerning due to its high aggressiveness to a wide range of tree species and its involvement in oak and beech declines across Europe (Jung 2009; Jung and Burgess 2009; Jung et al. 2018a).

The stream surveys in South Moravia and in Žilina revealed the presence of several aquatic *Phytophthora* species and also one terrestrial species, *P. syringae*, in one South Moravian stream. Seasonality of these oomycetes (cf. Hwang et al. 2008, 2011) is most likely responsible for the lower *Phytophthora* diversity in these regions as compared to Austria since the surveys in Czechia and Slovakia were performed only in November while in Austria the surveys were performed in different seasons (spring, summer and autumn). The high prevalence of *P. gonapodyides* and *P. lacustris* in watercourses of Central Europe is in accordance with previous surveys performed in the Mediterranean (Jung et al. 2019; Seddaiu et al. 2020; Riolo et al. 2020) and Northern Europe (Redondo et al. 2018a). Both species are considered native to Europe and widespread on several continents (Greslebin et al. 2005; Reeser et al. 2011; Huai et al. 2013; Nagel et al. 2013; Shrestha et al. 2013; Stamler et al. 2016; Jung et al. 2018b; Xu et al. 2019). *Phytophthora lacustris* is predominantly an aquatic saprotroph on plant debris but can also act as opportunistic pathogen of riparian trees and irrigated trees and crops (Orlikowski et al. 2011; Nechwatal et al. 2013; Jung et al. 2016; Kanoun-Boulé et al. 2016). Soil infestation assays with *P. lacustris* have also shown moderate aggressiveness to several tree species (Nechwatal et al. 2013; Milenković et al. 2018). In contrast, *P. gonapodyides* has a wider range of habitats than *P. lacustris* (Jung et al. 1996; Hansen and Delatour 1999; Reeser et al. 2011; Seddaiu et al. 2020) and besides being an aquatic saprotroph has also been reported as virulent root and bark pathogen on mature forest trees and in pathogenicity tests (Jung et al. 1996, 2000; Jung 2009; Corcobado et al. 2010, 2020; Belisario et al. 2016; Cleary et al. 2016). It is notable that *P. gonapodyides* could also be isolated during the coldest months as in the present and previous studies (Hansen and Delatour 1999; Hwang et al. 2011) demonstrating its ecological advantage to proliferate during all seasons. Moreover, *P. gonapodyides* showed the widest altitudinal amplitude (141.1–1292.8 m) and reached the highest altitudinal location surveyed in the Austrian Alps. Another clade 6 species, *P. bilorbang*, was only found in one river (2.7%) in Austria while it was present in two rivers (20%) surveyed in South Moravia and in Žilina. Also, in all three countries *P. bilorbang* was isolated only from fallen leaves floating on the waterbodies during autumn. This pathogen has been found previously in aquatic ecosystems in Italy and the USA (Reeser et al. 2011; Seddaiu et al. 2020) and is associated with declining maquis shrub species (i.e. *Juniperus phoenicea* and *Pistacia lentiscus*), riparian vegetation (i.e. *Salix* sp. and *A. glutinosa*) (Scanu et al. 2015; Riolo et al. 2020; Matsiakh et al. 2021) and with root rot in olive (*Olea europaea*) orchards (Santilli et al. 2020). *Phytophthora bilorbang* has very similar ITS sequences as *Phytophthora* taxon Oaksoil and *Phytophthora* taxon Riversoil, both informally designated in 2003 (Brasier et al. 2003). However, *P. bilorbang* has a homothallic breeding system whereas the other two taxa are sterile, considered as an adaptation to a fully aquatic saprotrophic lifestyle (Brasier et al. 2003; Jung et al. 2011). Interestingly, a previously unknown taxon closely related to *P. bilorbang*, informally designated *P.* taxon bilorbang-like2, was also isolated from floating leaves in Austria during autumn. Another common clade 6 species in the waterways of Austria and South Moravia was *P. riparia*, a widespread aquatic saprotroph in the Northern hemisphere which is often involved in hybridization processes (Brazee et al. 2016; Stamler et al. 2016; Bily et al. 2018, 2022), also demonstrated by the findings of the hybrids *P.* taxon × *riparia* and *P. lacustris* × *riparia* in Austrian and Czech streams, respectively. Inoculation tests with *P. riparia* and several crop species have demonstrated lack of pathogenicity (Stamler et al. 2016). *Phytophthora chlamydospora*, another clade 6 species, was exclusively detected in three Austrian watercourses. Considered as native in North America, *P. chlamydospora* has been found widespread in all continents except Antarctica (Greslebin et al. 2005; Jung 2009; Reeser et al. 2011; Huai et al. 2013; Oh et al. 2013; Hansen et al. 2015; Sims et al. 2015; Jung et al. 2016, 2017a, 2018b, 2019, 2020; Bily et al. 2018, 2022; Xu et al. 2019; Corcobado et al. 2020; Seddaiu et al. 2020). Although *P. chlamydospora* has a predominantly waterborne saprophytic lifestyle (Brasier et al. 2003; Hansen et al. 2015; Sims et al. 2015; Bily et al. 2018, 2022), it can act as an opportunistic tree pathogen in natural ecosystems (Bily et al. 2022) and as aggressive pathogen in orchards and nurseries (Derviş et al. 2016; Jung et al. 2016; Browne et al. 2020, 2021). In addition, a hybrid between *P. chlamydospora* and *P. lacustris*, *P. chlamydospora* × *lacustris*, was recovered from one Austrian river. *Phytophthora hydropathica* from a fast-growing, high-temperature tolerant cluster of species in clade 9 was only isolated once from the Danube river in Upper Austria. It is a typical aquatic saprotroph and opportunistic pathogen of crops which is widespread in the USA (Hong et al. 2010; Shrestha et al. 2013) and was recently found in streams in Spain, Sicily and Sardinia (Pintos et al. 2016; Jung et al. 2019; Seddaiu et al. 2020). It is expected that the activity of this species in Central Europe will increase with warming climate. The only species from Clade 10 recovered in this study was *P. gallica*, occurring in 30% of the streams surveyed in South Moravia. It has been documented inhabiting waterways and riparian terrestrial ecosystems, causing occasional damage as root pathogen (Jung and Nechwatal 2008; Sims et al. 2015; Redondo et al. 2018a; Christova 2022).

Interspecific hybridization is increasingly recognised as driving evolutionary force in the genus *Phytophthora* facilitating adaptation to new or changing environmental conditions and enlarging host ranges or enabling host jumps, occasionally leading to speciation (Brasier 2000; Bertier et al. 2013; Nagel et al. 2013; Burgess 2015; Jung et al. 2017a, b, 2018b; Van Poucke et al. 2021). Aquatic environments seem to be a particularly favourable habitat for interspecific hybridizations as demonstrated by an array of aquatic hybrids within clades 6, 7 and 9 (Nagel et al. 2013; Yang et al. 2014; Burgess 2015; Brazee et al. 2016; Jung et al. 2017a, b, 2018a). Also in the present study, several clade 6 hybrids were obtained from watercourses (i.e. *P. chlamydospora* × *lacustris*, *P.* taxon × *lacustris* and *P.* taxon × *riparia*).

*Nothophytophthora*, a sister genus of *Phytophthora* within the *Peronosporaceae*, was recently described (Jung et al. 2017c) and members from this genus have since been reported in waterbodies and terrestrial ecosystems (Jung et al. 2018a; O’Hanlon et al. 2021; Landa et al. 2021). Also in this study, two new *Nothophytophthora* taxa, informally designated as *Nothophytophthora* taxon 1 and *Nothophytophthora* taxon 2, were obtained from each one stream in Slovakia and Czechia, respectively. Other oomycete genera from the families of the *Pythiaceae* (*Elongisporangium*, *Pythium*) and the *Peronosporaceae (Halophytophthora*, *Phytopythium*) were frequently present but only a few were subcultured and sequenced as they were not the goal of this study. *Phytopythium litorale* was recovered from Central European waterbodies and has a global distribution (e. g. Choudhary et al. 2016; Jung et al. 2020). It has also been identified as an aggressive pathogen of Oriental plane trees (*Platanus orientalis*) in Turkey (Derviş et al. 2020). *Elongisporangium undulatum* was isolated from a baiting raft in the Danube. The species is known as soilborne fine root pathogen from natural ecosystems worldwide (Jung et al. 1996, 2000, 2017a, 2018b) and was less often reported from waterbodies (O'Hanlon et al. 2018). The genus *Halophytophthora* is closely related to *Phytophthora* and *Nothophytophthora* and comprises mainly species from marine and brackish-water habitats (Nigrelli and Thines 2013; Maia et al. 2022) but they can also tolerate low salinity and even inhabit freshwater ecosystems (Hüberli et al. 2013; Yang and Hong 2014). *Halophytophthora fluviatilis* was described from rivers and streams in the eastern USA (Yang and Hong 2014). It was also found widespread in rivers and streams in eastern Spain and its pathogenicity to different Fagaceae species and black alder was demonstrated (Caballol et al. 2021). In the present study, *H. fluviatilis* was recovered from naturally fallen autumn leaves in lowland rivers of Austria and Czechia, demonstrating its capacity to survive as saprotroph in freshwater environments.

The riparian alder forests surveyed in Austria showed an intermediate level of decline and low mortality rates. In Austria alder dieback associated with *Phytophthora* in Austria was first detected in 1996. Subsequently, an extensive alder survey was performed between 1996 and 1999 and the presence of the so-called ‘alder-*Phytophthora*’ was confirmed at a few scattered sites along the watercourses Reibach, Thaya, Kamp and Danube in Lower Austria and Upper Austria (Cech and Brandstetter 1999). A later alder survey performed in 50 sites in the state of Vienna showed a low percentage of mortality (3.74%) which was mostly associated with drought except for ten sites where collar rot cankers were detected and *Phytophthora* spp. confirmed as causal agents (Cech 2006). More extensive surveys revealed widespread occurrence of alder dieback in Austria, with higher disease incidences in Burgenland and Styria (Balci and Cech 2004). The assessments of the present study (2014–2019) demonstrated high mortality levels and chronic decline in the state of Styria and, to a lesser extent, in the states of Tyrol and Salzburg. In some parts of Styria, the decline was already extremely advanced with predominance of secondary pathogens, so that *Phytophthora* spp. could only rarely be isolated (i.e. along streams Turrach and Marbach). It was also noticed that these areas were associated with disturbances such as the construction of water channels which might have affected the water regime and vitality of alders. Since in these states several diseased alder plantings were found it is likely that *P.* × *alni* and other *Phytophthora* spp. were introduced with infested planting stock as demonstrated in previous studies (Jung and Blaschke 2004; Jung et al. 2016). Similar observations regarding infested nursery stock as potential pathway of *P.* × *alni* to natural riparian alder stands in Austria were made by Balci and Cech (2004). Other Central European countries, such as Germany, Czechia and Poland, also reported *Phytophthora*-related alder dieback during the 1990s (Hartmann 1995; Jung and Blaschke 2004) or the 2000s (Černý et al 2003; Nagy et al. 2003; Orlikowski et al. 2003; Černý et al. 2008). In these surveys the distribution of diseased alders was mainly observed in lowland forests while alpine riparian forests were either overlooked or not investigated in detail (Cech and Brandstetter 1999; Jung and Blaschke 2004). Later, monitoring of declining alder sites at alpine sites in Italy produced inconclusive results as symptoms but no primary agent were found (Pisetta et al. 2012). However, recently the presence of declining grey and green alders in the Italian Alps was found associated with *Phytophthora* infections (Bregant et al. 2020). This study recorded the presence of declining alders up to 1400 m altitude with *P. uniformis* being isolated from bark cankers and rhizosphere soil at altitudes of 1266 m and 1081.9 m, respectively. This is in accordance with results from the present study and with previous studies (Adams et al. 2008; Štěpánková et al. 2013; Redondo et al. 2015) which showed prevalence of *P. uniformis* at higher altitudes while *P.* × *alni* was exclusively found in the lowlands. In Italy, a variety of *Phytophthora* species, including the new species *P. alpina*, were isolated from rhizosphere and stem cankers from grey and green alder at elevations between 1412 and 1803 m, but remarkably no species from the *P.* × *alni* complex was found (Bregant et al. 2020).

This study revealed a diverse assemblage of nine *Phytophthora* species associated with riparian alder stands in Austria, i.e. *P. cactorum*, *P. gonapodyides*, *P. lacustris*, *P. plurivora*, *P. polonica*, *P. syringae*, *P. uniformis*, *P.* × *alni* and *P.* × *multiformis*, which were also recorded in previous alder surveys elsewhere (Jung and Blaschke 2004; Štěpánková et al. 2013; Jung et al. 2013, 2018a; Trzewik et al. 2015; Aday Kaya et al. 2018). In previous surveys performed in Austria, *P.* × *alni*, *P.* × *multiformis* and *P. plurivora* were isolated from bark cankers of alders in areas with common occurrence of alder plantations (Balci and Cech 2004; Cech 2006; Jung et al. 2013). Also in this study, the presence of *P.* × *multiformis* was related to a riparian *A. incana* plantation in Vienna state. It appears that the occurrence of *P.* × *multiformis* is often related to the recent introduction with infected nursery plants (Jung and Blaschke 2004; Štěpánková et al. 2013; Jung et al. 2016) which might be the reason for its infrequent occurrence in regions with rare alder planting activities (Nagy et al. 2003; Pintos et al. 2016). The most common *Phytophthora* species in Austrian alder stands in this study was *P. plurivora*. This wide-host range pathogen (previously designated as *P. citricola*; Jung & Burgess 2009) is widespread in the Northern hemisphere and one of the most important invasive pathogens in European forests. Due to its high aggressiveness *P. plurivora* is a major driver of declines and diebacks of beech and oak stands across Europe and is also responsible for the mortality of many alders and other tree species (Jung et al 1996, 2000, 2013, 2016, 2018a, 2019; Hansen and Delatour 1999; Jung and Blaschke 2004; Jung and Burgess 2009; Mrazkova et al. 2013; Trzewik et al. 2015; Seddaiu and Linaldeddu 2020; Corcobado et al. 2020; Matsiakh et al. 2021). Its pathogenicity to alders, beech and oaks has been demonstrated in numerous trials (Jung et al 1996; Fleischmann et al. 2002, 2004; Jung and Nechwatal 2008; Jung 2009; Zamora-Ballesteros et al. 2017; Corcobado et al. 2022). Although *P. plurivora* has been isolated previously from alder bark cankers in Austria, Bavaria and Italy (Jung and Blaschke 2004; Cech 2006; Bregant et al. 2020) it was recovered only from alder rhizosphere in the present study. The altitudinal range at which *P. plurivora* was found in this study was 144.5 to 793.7 m, confirming previous reports from beech and alder stands in Austria, Bavaria and Czech Republic (Jung and Blaschke 2004; Mrázková et al. 2013; Corcobado et al. 2020). In contrast, in the Italian Alps *P. plurivora* has been isolated from alder bark cankers and from rhizosphere at an altitude of 1412 m (Bregant et al. 2020). In one Austrian alder stand *P. plurivora* co-occurred in rhizosphere soil with *P. syringae* which has previously been found associated with alders as a weak pathogen (Trzewik et al. 2015). Isolation tests from alder fine roots yielded exclusively *P. polonica* and *P. lacustris*. *Phytophthora polonica* is considered native to Europe and has been mainly isolated from rhizosphere soil in declining riparian alder forests (Belbahri et al. 2006; Matsiakh et al. 2021), declining poplar plantations (Milenković et al. 2018) and declining *Q. robur* forests (Jankowiak et al. 2014). Soil infestation tests demonstrated its high aggressiveness to poplar roots (Milenković et al. 2018) while under-bark inoculation tests showed only mild pathogenicity to poplar and oak (Jankowiak et al. 2014; Milenković et al. 2018). In addition, the clade 6 species *P. lacustris* and *P. gonapodyides* were isolated from fine roots and rhizosphere of both declining and non-declining alders. Although being dominant species in waterways with proven pathogenicity to alders (Navarro et al. 2015; Trzewik et al. 2015) they are only rarely recorded from declining alders (Jung and Blaschke 2004; Černý et al. 2011; Sims et al. 2015; Trzewik et al. 2015; Bregant et al. 2020).

Continuous studies of *Phytophthora* diversity in the waterways of Europe are revealing almost ubiquitous infestations with an increasing number of *Phytophthora* species and hybrids and also other oomycetes such as *Nothophytophthora* and *Halophytophthora* spp.. Further studies are needed to clarify their actual distribution and their ecological roles. Due to the wide host ranges of some *Phytophthora* species, e.g. *P. cactorum* and *P. plurivora*, and the largely unknown host ranges of most *Phytophthora* species and hybrids surface water should not be used for irrigation of trees and crops. Extensive host range studies on major tree and crop species should be performed to unveil the potential threat posed by the *Phytophthora*, *Nothophytophthora* and *Halophytophthora* taxa found in Central European rivers and streams. This study also showed that *P. plurivora*, *P. uniformis*, *P.* × *alni* and *P.* × *multiformis* were the main causal agents in the aetiology of alder decline in Austria. Although alder cankers in alpine areas were exclusively caused by *P. uniformis*, *P. plurivora* proved to be the most widespread oomycete in riparian alder forests. Due to its aggressiveness to alders *P. plurivora* might pose an increasing threat to alder stands in the future.

## Supplementary Information

Below is the link to the electronic supplementary material.

Supplementary file1 (PDF 181 kb)

Supplementary file2 (PDF 183 kb)

Supplementary file3 (PDF 144 kb)

Supplementary file4 (PDF 1182 kb)

Supplementary file5 (PDF 762 kb)

Supplementary file6 (PDF 1106 kb)

Supplementary file7 (PDF 304 kb)

Supplementary file8 (PDF 938 kb)

Supplementary file9 (PDF 766 kb)

Supplementary file10 (PDF 945 kb)

Supplementary file11 (PDF 1656 kb)

Supplementary file12 (PDF 1024 kb)

Supplementary file13 (PDF 861 kb)

## Data Availability

The DNA sequences generated during the current study are available in Genbank.
