# Supplementary material for: Phytophthora, Nothophytophthora and Halophytophthora diversity in rivers, streams and riparian alder ecosystems of Central Europe
Source: Mycol Prog. 2023 Jun 13;22(7):50. doi: 10.1007/s11557-023-01898-1 (PMC10264269; doi:10.1007/s11557-023-01898-1)
Supplement: Supplementary file 1 — Supplementary file1 (PDF 181 kb) [file 11557_2023_1898_MOESM1_ESM.pdf]

Phytophthora, *Nothophytophthora* and *Halophytophthora* diversity in rivers, streams and riparian alder ecosystems of Central Europe

Tamara Corcobado[1,2], Thomas L. Cech[2], Andreas Daxer[2], Henrieta Ďatková[1], Josef Janoušek[1], Sneha Patra[1,3], Daniella Jahn[2], Christine Hüttler[2], Ivan Milenković[1,4], Michal Tomšovský[1], Marília Horta Jung[1,5], Thomas Jung[1,5]*

[1]Phytophthora Research Centre, Faculty of Forestry and Wood Technology, Mendel University in Brno, Zemědělská 3, 61300 Brno, Czech Republic; tamara.corcobado@mendelu.cz (T.C.); xdatkova@mendelu.cz (H.D.); janousek.jose@gmail.com (J.J.); (I.M) marilia.jung@mendelu.cz (M.H.J.); ivan.milenkovic@mendelu.cz (I.M.); michal.tomsovsky@mendelu.cz (M.T.)

[2]Federal Research and Training Centre for Forests, Natural Hazards and Landscape, Unit of Phytopathology, Department of Forest Protection, Seckendorff-Gudent-Weg 8, 1131 Vienna, Austria. tamara.corcobado@bfw.gv.at (T.C.); thomas.cech@bfw.gv.at (T.L.C.); andreas.daxer@bfw.gv.at (A.D.); daniela.jahn13@gmail.com (D.J); christine.huettler@bfw.gv.at (C.H.).

[3]Global Change Research Institute of the Czech Academy of Sciences, Belidla 986/4a, 603 00 Brno, Czech Republic; snehampatra@gmail.com (S.P.)

[4]University of Belgrade, Faculty of Forestry, Kneza Višeslava 1, 11030 Belgrade, Serbia;

[5]Phytophthora Research and Consultancy, Am Rain 9, 83131 Nußdorf, Germany.

Table S1. *Phytophthora*, *Halophytophthora* and *Nothophytophthora* taxa isolated between 2014 and 2019 from 68 watercourses and 151 sampling sites in Austria, the Czech Republic and Slovakia using six sampling techniques.

River no.	River, state	Sample type, host species (year)	*Phytophthora*, *Halophytophthora* and *Nothophytophthora* spp. (site codes) [a, b]
R01	Alvier, Seebach, tributary of river Ill; Vorarlberg	baiting raft (2014)	0 (39)
R02	Danube, Lower Austria, Upper Austria	Bark canker *A. incana* (2014)	×MUL (**4**)
		Baiting raft (2014)	HYD, LAC (**4**)
		Fine roots (2014)	LAC (**4**)
		Baiting raft (2014)	GON, LAC (5)
		Water filtration (2015)	0 (6)
		Baiting raft (2015)	LAC (7)
		Baiting raft (2015)	LAC (8)
		Baiting raft (2015)	LAC (9)
		Baiting raft (2015)	0 (10)
		Water filtration (2016)	0 (1)
		Water filtration (2016)	LAC, RIP (2)
		Water filtration (2016)	LAC (3)
R03	Drau, tributary of river Danube; Salzburg	Baiting raft (2015)	GON, LAC (15)
		Baiting raft (2015)	0 (16)
		water filtration (2015)	0 (16)
		Rhizosphere soil *A. incana, A. glutinosa* (2019)	GON, LAC (**12**)
		Floating fallen leaves (2019)	GON (13)
R04	Einödgrabenbach, tributary of river Drau; Salzburg	Floating fallen leaves (2019)	0 (14)
R05	Enns, tributary of river Danube; Styria	Bark canker *A. incana* (2015)	0 (19)
		Bark canker *A. incana* (2015)	UNI (20)
		Rhizosphere soil *A. incana* (2015)	PLU (20)
		Rhizosphere soil *A. incana* (2015)	0 (21)
		Baiting raft (2015)	×CHL, LAC (22)
		Baiting raft (2015)	LAC (23)
		Baiting raft (2015)	GON, LAC, PLU (24)
		Baiting raft (2015)	GON, LAC, PLU (25)
		Water filtration (2015)	0 (26)
		Rhizosphere soil *A. incana* (2019)	PLU, SYR (27)
		Floating fallen leaves (2019)	GON, LAC (27)
R06	Fugnitz, tributary of river Thaya; Lower Austria	Rhizosphere soil *A. glutinosa* (2015)	0 (117)
		Rhizosphere soil *A. glutinosa* (2015)	0 (118)
R07	Gschnitzbach, tributary of Inn; Tyrol	Baiting raft (2014)	0 (46)
		Rhizosphere soil *A. incana* (2014)	0 (46)
		Rhizosphere soil *A. incana* (2014)	UNI (49)
		Bark canker *A. incana* (2014)	0 (49)
		Bark canker *A. incana* (2014)	0 (**51**)
		Bark canker *A. incana* (2014)	0 (52)
		Bark canker *A. incana* (2014)	0 (53)

River no.	River, state	Sample type, host species (year)	***Phytophthora*, *Halophytophthora* and *Nothophytophthora* spp. (site codes)** [a, b]
R08	Gurk, tributary of Drau; Carinthia	Baiting raft (2015)<br>Baiting raft (2015)	GON (29)<br>0 (30)
R09	Ill, Voralberg	Baiting raft (2014)<br>Baiting raft (2014)	0 (41)<br>0 (42)
R10	Inn, tributary of river Danube; Upper Austria, Tyrol	Baiting raft (2014)<br>Baiting raft (2014)<br>Rhizosphere soil *A. incana* (2014)<br>Rhizosphere soil *A. incana* (2014)<br>Bark canker *A. incana* (2014)<br>Bark canker *A. incana* (2014)<br>Water filtration (2015)<br>Water filtration (2015)<br>Water filtration (2016)	GON, LAC, UNI (47)<br>0 (48)<br>GON (48)<br>PLU (50)<br>×ALN (50)<br>UNI (54)<br>GON, LAC (43)<br>GON (44)<br>LAC (45)
R11	Isel, tributary of river Drau; Tyrol	Rhizosphere soil *A. incana* (2014)<br>Bark canker *A. incana* (2014)	PLU (11)<br>0 (11)
R12	Jaudlingbach, tributary of river Thaya; Lower Austria	Rhizosphere soil *A. incana*	PLU (121)
R13	Kamp,tributary of Danube; Lower Austria	baiting raft (2014)<br>Rhizosphere soil *A. glutinosa* (2014)<br>Rhizosphere soil *A. glutinosa* (2014)<br>Rhizosphere soil *A. glutinosa* (2014)<br>Water filtration (2015)<br>Rhizosphere soil *A. glutinosa* (2015)<br>Rhizosphere soil *A. glutinosa* (2015)<br>Water filtration (2016)<br>Rhizosphere soil *A. glutinosa* (2019)<br>Floating fallen leaves (2019)	LAC, PLU, ×RIP (**55**)<br>0 (**55**)<br>0 (56)<br>0 (57)<br>0 (58)<br>0 (58)<br>0 (59)<br>LAC (60)<br>PLU (61)<br>BIL, FLU, GON, LAC (61)
R14	Krumme Steyerling, tributary of river Enns; Upper Austria	Baiting raft (2015)<br>Rhizosphere soil riparian *A. incana, Populus sp.* and *Salix* sp. (2015)<br>Baiting raft (2015)	CHL, GON, LAC, PLU, ×RIP (31)<br>CAC (31)<br>GON (32)
R15	Lafnitz, tributary of river Raab; Burgenland	Baiting raft (2014)<br>Rhizosphere soil *A. glutinosa* (2014)	LAC (**100**)<br>PLU (**100**)
R16	Lainsitz, tributary of Elbe; Lower Austria	Rhizosphere soil *A. glutinosa* (2015)	PLU (120)
R17	Lammer, tributary of river Salzach; Salzburg	Floating fallen leaves (2019)	GON, LAC (104)
R18	Laue, tributary of river Drau; Tyrol	Rhizosphere soil *A. incana* (2014)<br>Bark canker *A. incana* (2014)	0 (18)<br>0 (18)
R19	Leutascher Ache, tributary of river Danube; Tyrol	Baiting raft (2015)<br>Baiting raft (2015)	GON (33)<br>0 (34)
R20	Liesing, tributary of river Mur; Styria	Baiting raft (2015)<br>Rhizosphere soil *A. glutinosa* (2015)<br>Baiting raft (2015)	GON (35)<br>PLU (35)<br>CHL, GON (36)
R21	Lonka; tributary of river Mur; Salzburg	Baiting raft (2014)	0 (91)
R22	Lungitzer Loben, tributary of river Lafnitz; Styria	Rhizosphere soil *A. glutinosa* (2014)<br>Bark canker soil *A. glutinosa* (2014)<br>Baiting raft (2014)<br>Fine roots soil *A. glutinosa* (2015)	0 (**131**)<br>×ALN (**131**)<br>GON, LAC, PLU (**131**)<br>POL (**131**)
R23	Lünitzbach, tributary of river Möll; Carinthia	Floating leaves (2019)	GON, LAC (75)
R24	Lutz, Lägerzunbach, tributary of river Ill; Voralberg	Baiting raft (2014)	LAC (40)
R25	Marbach, tributary of river Danube; Upper Austria	Bark canker soil *A. glutinosa* (2014)<br>Fine roots soil *A. glutinosa* (2015)	×ALN, UNI (**129**)<br>0 (**129**)
R26	March, tributary of river Danube; Lower Austria	Rhizosphere soil *A. glutinosa* (2014)<br>baiting raft (2014)<br>Water filtration (2015)<br>Water filtration (2016)<br>Water filtration (2016)	0 (**63**)<br>LAC (**63**)<br>LAC (65)<br>LAC (66)<br>LAC (67)
R26	March, tributary of river Danube; Lower Austria	Floating leaves (2019)<br>Rhizosphere soil *A. glutinosa* (2019)<br>Floating fallen leaves (2019)<br>Rhizosphere soil *A. glutinosa* (2019)	BIL2, LAC (68)<br>0 (68)<br>GON, LAC (69)<br>PLU (69)

River no.	River, state	Sample type, host species (year)	*Phytophthora*, *Halophytophthora* and *Nothophytophthora* spp. (site codes) [a, b]
R27	Möll, tributary of river Drau; Carinthia	Baiting raft (2014)<br>Rhizosphere soil *A. incana* (2015)<br>Water filtration (2015)<br>Bark canker *A. incana* (2015)<br>Bark canker *A. incana* (2015)<br>Floating fallen leaves (2019)<br>Rhizosphere soil *A. glutinosa* (2019)<br>Floating fallen leaves (2019)<br>Baiting raft (2019)	0 (72)<br>0 (76)<br>0 (77)<br>0 (78)<br>0 (79)<br>GON, LAC (73)<br>0 (73)<br>0 (74)<br>LAC (77)
R28	Mur, tributary of river Danube; Salzburg, Styria	Baiting raft (2014)<br>Baiting raft (2014)<br>Baiting raft (2014)<br>Bark canker *A. incana* (2014)<br>Baiting raft (2014)<br>Water filtration (2015)<br>Water filtration (2016)<br>Water filtration (2016)<br>Floating fallen leaves (2019)<br>Rhizosphere soil *A. glutinosa* (2019)	0 (**84**)<br>0 (86)<br>GON (88)<br>0 (89)<br>0 (**90**)<br>0 (81)<br>LAC (82)<br>GON, LAC (83)<br>GON, LAC, ×RIP (93)<br>0 (95)
R29	Raab, tributary of river Danube; Styria, Burgerland	Baiting raft (2014)<br>Rhizosphere soil *A. glutinosa* (2014)<br>Water filtration (2015)<br>Water filtration (2016)<br>Water filtration (2016)	LAC (**99**)<br>0 (**99**)<br>LAC (96)<br>0 (97)<br>CHL, LAC, PLU (98)
R30	Raggabach, tributary of river Möll; Carinthia	Bark canker *A. incana* (2015)	0 (80)
R31	Reißbach, tributary of river Lainsitz; Lower Austria	Bark canker soil *A. glutinosa* (2014)<br>Fine roots soil *A. glutinosa* (2015)	×ALN, ×MUL (**128**)<br>0 (**128**)
R32	Riegersburgerbach, tributary of river Thaya; Lower Austria	Water filtration (2015)	0 (119)
R33	Rosenbach, tributary of river Drau; Carinthia	Baiting raft (2015)	0 (17)
R34	Rötzbach, tributary of river Mur; Styria	Floating fallen leaves (2019)	GON, LAC (94)
R35	Salzach, tributary of river Danube; Salzburg, Upper Austria	Baiting raft (2014)<br>Baiting raft (2014)<br>Baiting raft (2014)<br>Bark canker *A. incana* (2014)<br>Rhizosphere soil *A. incana* (2014)<br>Bark canker *A. incana* (2014)<br>Bark canker *A. incana* (2014)<br>Rhizosphere soil *A. incana* (2014)<br>Water filtration (2015)<br>Water filtration (2016)<br>Floating fallen leaves (2019)<br>Rhizosphere soil *A. glutinosa* (2019)	0 (106)<br>0 (107)<br>GON (**108**)<br>×ALN (**109**)<br>PLU (**109**)<br>0 (110)<br>0 (**111**)<br>0 (**111**)<br>GON (101)<br>GON, LAC (102)<br>0 (103)<br>GON (105)
R36	Sipbach, tributary of river Traun; Upper Austria	Fine roots soil *A. glutinosa* (2015)	0 (130)
R37	Stempfelbach, tributary of river March; Lower Austria	Floating fallen leaves (2019)	BIL, GON, LAC, ×LAC (70)
R38	Stiefernbach, tributary of river Kamp; Lower Austria	Floating fallen leaves (2019)	BIL, GON, LAC (62)
R39	Thaya, tributary of river March; Lower Austria	Water filtration (2015)<br>Baiting raft (2015)<br>Rhizosphere soil *A. glutinosa* (2015)<br>Water filtration (2015)<br>Water filtration (2016)<br>Water filtration (2016)	0 (114)<br>LAC (115)<br>0 (116)<br>LAC (116)<br>LAC (112)<br>LAC, ×RIP (113)
R40	Thomatalerbach, tributary of river Mur; Salzburg	Baiting raft (2014)	GON (92)
R41	Traun, tributary of river Danube; Upper Austria	Baiting raft (2014)<br>Rhizosphere soil *A. incana* (2014)<br>Baiting raft (2014)<br>Rhizosphere soil *A. incana* (2014)<br>Rhizosphere soil *A. glutinosa* (2014)<br>Water filtration (2015)<br>Floating fallen leaves (2019)	GON, LAC (**122**)<br>0 (**122**)<br>RIP (**123**)<br>0 (**123**)<br>PLU (124)<br>LAC, PLU (125)<br>GON, LAC (126)
R42	Turrach, tributary of river Mur; Styria	Bark canker *A. incana* (2014)	UNI (87)

River no.	River, state	Sample type, host species (year)	*Phytophthora*, *Halophytophthora* and *Nothophytophthora* spp. (site codes) [a, b]
R43	Weidenbach, tributary of river March; Lower Austria	Rhizosphere soil *A. glutinosa* (2019)	PLU (71)
R44	Weißenbach, tributary of river Enns; Styria	Floating fallen leaves (2019)	GON (28)
R45	Weizbach, tributary of river Raab; Styria	Baiting raft (2015)<br>Rhizosphere soil riparian *A. glutinosa* , *Populus* sp. and *Salix* sp. (2015)<br>Rhizosphere soil riparian *Alnus* sp and *Salix* sp. (2015)	GON, PLU (37)<br>LAC, PLU (37)<br>0 (38)
R46	Wölzerbach, tributary of river Mur; Styria	Baiting raft (2014)	0 (85)
R47	Zapfengraben, tributary of river March; Lower Austria	Baiting raft (2014)	LAC (64)
R48	Zeller Ache, Ager, tributary of river Traun; Upper Austria	Floating fallen leaves (2019)<br>Rhizosphere soil A. glutinosa (2019)	GON, LAC, ×LAC, ×RIP (127)<br>PLU (127)
R49	Šebrovka, tributary of river Danube; South Moravia	Floating fallen leaves (2018)	GON, LAC (132)
R50	Punkva, tributary of river Danube; South Moravia	Floating fallen leaves (2018)	BIL, FLU, GAL, GON, LAC, SYR, NOT2 (133)
R51	Kuřimka, tributary of river Danube; South Moravia	Floating fallen leaves (2018)	GON, LAC, BIL, GAL, RIP, ×LAC (134)
R52	Bílý Potok, tributary of river Danube; South Moravia	Floating fallen leaves (2018)	GON, LAC (135)
R53	Habřina, tributary of river Danube; South Moravia	Floating fallen leaves (2018)	GON, LAC (136)
R54	Troubský potok x Bobrava, tributary of river Danube; South Moravia	Floating fallen leaves (2018)	GON, LAC (137)
R55	Časnýř, tributary of river Danube; South Moravia	Floating fallen leaves (2018)	FLU, GON, LAC (138)
R56	Říčka, tributary of river Danube; South Moravia	Floating fallen leaves (2018)	GAL, GON, LAC (139)
R57	Rakovec, tributary of river Danube; South Moravia	Floating fallen leaves (2018)	GON, LAC (140)
R58	Bílá voda, tributary of river Danube; South Moravia	Floating fallen leaves (2018)	GON, LAC (141)
R59	Črchľový potok,tributary of river Danube; Žilina	Floating fallen leaves (2018)	BIL, GON, LAC (142)
R60	Trstená, tributary of river Danube; Žilina	Floating fallen leaves (2018)	GON, LAC (143)
R61	Predmieranka, tributary of river Danube; Kysuce	Floating fallen leaves (2018)	BIL, GON, LAC (144)
R62	Unnamed tributary of Predmieranka; Žilina	Floating fallen leaves (2018)	GON (145)
R63	No name, tributary of river Danube; Žilina	Floating fallen leaves (2018)	GON (146)
R64	Ľalikov potok, tributary of river Danube; Žilina	Floating fallen leaves (2018)	0 (147)
R65	Hanzľov potok, tributary of river Danube; Žilina	Floating fallen leaves (2018)	GON (148)
R66	No name, tributary of river Danube; Žilina	Floating fallen leaves (2018)	GON (149)
R67	No name, tributary of river Danube; Žilina	Floating fallen leaves (2018)	GON (150)
R68	No name, tributary of river Danube; Žilina	Floating fallen leaves (2018)	NOT1 (151)

[a] ×ALN = *P. ×alni*, BIL = *P. bilorbang*, BIL2 = *P.* taxon bilorbang-like 2, CAC = *P. cactorum*, CHL = *P. chlamydospora*, ×CHL = *P. chlamydospora × lacustris*, FLU = *Halophytophthora fluviatilis*, GAL = *P. gallica*, GON = *P. gonapodyides*, HYD = *P. hydropathica*, LAC = *P. lacustris*, ×LAC = *P.* taxon ×*lacustris*, ×MUL = *P. ×multiformis*, NOT1 = *Nothophytophthora* taxon 1, NOT2 = *Nothophytophthora* taxon 2, PLU = *P. plurivora*, POL = *P. polonica*, RIP = *P. riparia*, ×RIP = *P.* taxon ×riparia, SYR = *P. syringae*, UNI = *P. uniformis*.
[b] Site codes in bold refer to the 17 riparian sites which were assessed more in detail.
