# Supplementary material for: Phytophthora, Nothophytophthora and Halophytophthora diversity in rivers, streams and riparian alder ecosystems of Central Europe
Source: Mycol Prog. 2023 Jun 13;22(7):50. doi: 10.1007/s11557-023-01898-1 (PMC10264269; doi:10.1007/s11557-023-01898-1)
Supplement: Supplementary file 2 — Supplementary file2 (PDF 183 kb) [file 11557_2023_1898_MOESM2_ESM.pdf]

Phytophthora, *Nothophytophthora* and *Halophytophthora* diversity in rivers, streams and riparian alder ecosystems of Central Europe

Tamara Corcobado[1,2], Thomas L. Cech[2], Andreas Daxer[2], Henrieta Ďatková[1], Josef Janoušek[1], Sneha Patra[1,3], Daniella Jahn[2], Christine Hüttler[2], Ivan Milenković[1,4], Michal Tomšovský[1], Marília Horta Jung[1,5], Thomas Jung[1,5]*

[1]Phytophthora Research Centre, Faculty of Forestry and Wood Technology, Mendel University in Brno, Zemědělská 3, 61300 Brno, Czech Republic; tamara.corcobado@mendelu.cz (T.C.); xdatkova@mendelu.cz (H.D.); janousek.jose@gmail.com (J.J.); (I.M) marilia.jung@mendelu.cz (M.H.J.); ivan.milenkovic@mendelu.cz (I.M.); michal.tomsovsky@mendelu.cz (M.T.)

[2]Federal Research and Training Centre for Forests, Natural Hazards and Landscape, Unit of Phytopathology, Department of Forest Protection, Seckendorff-Gudent-Weg 8, 1131 Vienna, Austria. tamara.corcobado@bfw.gv.at (T.C.); thomas.cech@bfw.gv.at (T.L.C.); andreas.daxer@bfw.gv.at (A.D.); daniela.jahn13@gmail.com (D.J); christine.huettler@bfw.gv.at (C.H.).

[3]Global Change Research Institute of the Czech Academy of Sciences, Belidla 986/4a, 603 00 Brno, Czech Republic; snehampatra@gmail.com (S.P.)

[4]University of Belgrade, Faculty of Forestry, Kneza Višeslava 1, 11030 Belgrade, Serbia;

[5]Phytophthora Research and Consultancy, Am Rain 9, 83131 Nußdorf, Germany.

Table S2. Geographic coordinates and altitudes of 151 riparian sites sampled in the oomycete surveys in Austria, the Czech Republic and Slovakia 2014-2019.

Site no.	Latitude	Longitude	Altitude (m a.s.l)
1	48.34459	16.30595	167.0
2	48.33668	16.02764	181.1
3	48.40146	15.62440	195.8
4	48.13617	16.62988	149.3
5	48.27486	15.39158	202.0
6	48.39626	15.56654	197.7
7	48.46724	13.79484	282.4
8	48.32885	14.17158	253.2
9	48.21533	14.85563	234.8
10	48.17223	14.70331	232.8
11	46.83281	12.75865	643.3
12	47.77610	13.51250	528.7
13	47.77500	13.51780	525.0
14	47.77420	13.51750	545.8
15	46.54802	14.08884	461.4
16	46.81996	13.40810	544.8
17	46.55035	14.08060	461.3
18	46.80703	12.85383	645.0
19	47.39024	13.66492	744.4
20	47.45795	13.98079	659.2
21	47.46284	13.98381	658.1
22	47.92457	14.43360	332.1
23	47.71272	14.63694	405.0
24	47.57898	14.37114	624.9
25	47.42077	13.82077	690.8
26	47.42981	13.90495	673.3
27	47.55557	14.20740	632.6
28	47.55610	14.20720	634.5
29	46.82743	14.07710	775.4
30	46.81678	14.07490	800.4
31	47.84550	14.35432	503.4
32	47.85277	14.34122	487.3
33	47.38053	11.17195	1096.7
34	47.36302	11.15158	1174.2
35	47.43505	14.70000	793.7
36	47.42393	14.74850	747.2
37	47.24603	15.59701	526.6

Site no.	Latitude	Longitude	Altitude (m a.s.l)
38	47.23140	15.61076	490.2
39	47.09901	9.73748	1037.2
40	47.19783	9.77661	558.9
41	47.19735	9.69103	492.1
42	47.10922	9.86265	614.9
43	48.26133	13.03971	340.0
44	47.30005	11.06420	622.2
45	48.20551	12.93650	351.5
46	47.07407	11.40924	1197.8
47	47.48038	11.98223	507.7
48	47.20248	10.67612	730.9
49	47.08628	11.45074	1081.9
50	47.48038	11.98223	507.7
51	47.01789	11.31250	1408.7
52	47.02859	11.33007	1281.2
53	47.06861	11.39494	1198.5
54	47.46030	11.93205	511.3
55	48.57725	15.65958	240.9
56	48.57769	15.65931	242.0
57	48.63278	15.62633	262.4
58	48.47405	15.69908	204.6
59	48.48299	15.69615	207.9
60	48.42277	15.71507	196.1
61	48.58940	15.65610	248.0
62	48.53610	15.68170	234.3
63	48.34387	16.83806	143.5
64	48.26314	16.91122	140.4
65	48.35398	16.84218	141.1
66	48.23189	16.94809	140.0
67	48.60137	16.93291	151.1
68	48.24190	16.94530	140.4
69	48.36500	16.83810	146.7
70	48.20330	16.91860	141.1
71	48.34750	16.83530	144.5
72	46.85680	12.94561	832.5
73	46.87970	13.29080	616.9
74	46.90060	13.25420	667.9
75	46.87470	13.30030	689.2
76	46.93004	13.11942	698.9
77	46.93460	13.17174	674.5
78	46.93270	13.12820	693.4
79	46.87910	13.01252	794.4
80	46.92943	13.14011	740.3
81	47.08239	13.59389	1060.7
82	47.27076	15.32761	420.1
83	47.32250	14.97535	574.1
84	47.09346	13.63676	1043.3
85	47.15173	14.37207	743.9
86	47.21161	14.49833	712.1
87	46.96356	13.88577	1266.3
88	47.10628	13.47536	1137.0
89	47.10586	13.47597	1137.2
90	47.08753	13.68970	1029.6
91	47.18110	13.70010	1101.9
92	47.04590	13.71050	1292.8
93	47.40940	15.19890	492.0
94	47.39390	15.20720	560.9
95	47.26310	15.31440	424.7
96	46.95001	15.94151	269.6
97	47.13765	15.67149	367.3

Site no.	Latitude	Longitude	Altitude (m a.s.l)
98	46.97394	15.81025	296.9
99	46.92697	16.14407	239.7
100	46.99397	16.16961	232.8
101	47.94461	12.93645	391.4
102	47.83832	13.02108	405.3
103	47.65440	13.12140	452.7
104	47.58360	13.18000	474.6
105	47.54140	13.17060	495.8
106	48.06852	12.76841	371.6
107	47.67658	13.09908	442.5
108	47.28276	12.53859	780.3
109	47.28306	12.52712	782.4
110	47.27444	12.38179	808.8
111	47.37837	13.21502	553.8
112	48.73622	16.34903	176.9
113	48.85489	15.85871	287.0
114	48.91350	15.32580	450.3
115	48.89578	15.39775	432.0
116	48.90253	15.31947	450.9
117	48.80221	15.79499	405.5
118	48.81862	15.82511	372.9
119	48.82659	15.80558	371.7
120	48.71023	14.89735	512.3
121	48.82815	15.19099	559.2
122	47.56158	13.72117	525.1
123	48.21461	14.24708	266.9
124	48.21536	14.25044	265.4
125	48.08670	13.89685	337.1
126	48.09030	13.91890	332.1
127	48.08640	13.84720	348.1
128	48.90970	15.04040	504.3
129	48.28420	14.53980	311.8
130	48.18250	14.20970	293.1
131	47.32360	16.04690	369.1
132	49.33006	16.60538	338.0
133	49.36421	16.70364	354.0
134	49.28798	16.46251	242.0
135	49.27046	16.42509	259.0
136	49.18782	16.34342	343.0
137	49.13422	16.52900	235.0
138	49.25789	16.70151	286.0
139	49.24944	16.76291	353.0
140	49.28660	16.87480	326.0
141	49.40729	16.77936	466.0
142	49.43641	18.74828	444.0
143	49.47728	18.69761	524.0
144	49.49956	18.56346	775.0
145	49.49885	18.56353	775.0
146	49.44206	18.88161	498.0
147	49.43413	18.95549	651.0
148	49.45162	18.92685	586.0
149	49.46141	18.85653	611.0
150	49.47714	18.86866	657.0
151	49.47517	18.85867	619.0
