# Supplementary material for: Phytophthora, Nothophytophthora and Halophytophthora diversity in rivers, streams and riparian alder ecosystems of Central Europe
Source: Mycol Prog. 2023 Jun 13;22(7):50. doi: 10.1007/s11557-023-01898-1 (PMC10264269; doi:10.1007/s11557-023-01898-1)
Supplement: Supplementary file 3 — Supplementary file3 (PDF 144 kb) [file 11557_2023_1898_MOESM3_ESM.pdf]

Phytophthora, *Nothophytophthora* and *Halophytophthora* diversity in rivers, streams and riparian alder ecosystems of Central Europe

Tamara Corcobado[1,2], Thomas L. Cech[2], Andreas Daxer[2], Henrieta Ďatková[1], Josef Janoušek[1], Sneha Patra[1,3], Daniella Jahn[2], Christine Hüttler[2], Ivan Milenković[1,4], Michal Tomšovský[1], Marília Horta Jung[1,5], Thomas Jung[1,5*]

[1]Phytophthora Research Centre, Faculty of Forestry and Wood Technology, Mendel University in Brno, Zemědělská 3, 61300 Brno, Czech Republic; tamara.corcobado@mendelu.cz (T.C.); xdatkova@mendelu.cz (H.D.); janousek.jose@gmail.com (J.J.); (I.M) marilia.jung@mendelu.cz (M.H.J.); ivan.milenkovic@mendelu.cz (I.M.); michal.tomsovsky@mendelu.cz (M.T.)

[2]Federal Research and Training Centre for Forests, Natural Hazards and Landscape, Unit of Phytopathology, Department of Forest Protection, Seckendorff-Gudent-Weg 8, 1131 Vienna, Austria. tamara.corcobado@bfw.gv.at (T.C.); thomas.cech@bfw.gv.at (T.L.C.); andreas.daxer@bfw.gv.at (A.D.); daniela.jahn13@gmail.com (D.J); christine.huettler@bfw.gv.at (C.H.).

[3]Global Change Research Institute of the Czech Academy of Sciences, Belidla 986/4a, 603 00 Brno, Czech Republic; snehampatra@gmail.com (S.P.)

[4]University of Belgrade, Faculty of Forestry, Kneza Višeslava 1, 11030 Belgrade, Serbia;

[5]Phytophthora Research and Consultancy, Am Rain 9, 83131 Nußdorf, Germany.

Table S3. GenBank sequence accession numbers for DNA sequences of representative oomycete isolates obtained in the surveys in Austria (AU), the Czech Republic (CZ) and Slovakia (SK) 2014-2019.

Country	Oomycete species	Isolate code	ITS	*coxI*	*ß-tub*
AU	*Phytophthora bilorbang*	AU217	OP910274		
AU	*P. cactorum*	Hs1	OP910275		
AU	*P. chlamydospora*	Hr17	OP910276		
AU	*P. gonapodyides*	AU154	OP910277	OP903791	
AU	*P. lacustris*	AU124	OP910278	OP903792	
AU	*P. plurivora*	AU332	OP910279		
AU	*P. polonica*	R01	OP910280	OP903793	
AU	*P.* taxon bilorbang-like 2	AU199	OP910281	OP903794	
AU	*P. syringae*	AU353	OP910282		
AU	*P. uniformis*	B1.1	OP910283		
AU	*P. ×alni*	31	OP910284		
AU	*P. chlamydospora × lacustris*	R14	OP910285	OP903795	OP903821
AU	*P.* taxon ×*lacustris*	AU183	OP910286	OP903796	OP903822
AU	*P. ×multiformis*	33a	OP910287		
AU	*P.* taxon ×*riparia*	AU184	OP910288	OP903797	OP903823
AU	*Elongisporangium undulatum*	101	OP910300		
AU	*Halophytophthora fluviatilis*	AU221	OP910301		
AU	*Phytopythium litorale*	F36	OP910302		
AU	*Phy.* sp. PV_So7	84	OP910303		
AU	*Pythium* sp. 1 MNS-2013	75	OP910304		
AU	*Py.* sp. strain 1-9	F35	OP910305		
CZ	*P. bilorbang*	MO045	OP910289	OP903798	
CZ	*P. gallica*	MO130	OP910290	OP903799	
CZ	*P. gonapodyides*	MO011	OP910291	OP903800	
CZ	*P. lacustris*	MO016	OP910292	OP903801	
CZ	*P. syringae*	MO039	OP910293	OP903802	
CZ	*P.* taxon ×*lacustris*	MO136	OP910294	OP903803	OP903824
CZ	*Halophytophthora fluviatilis*	MO046	OP910306	OP903809	
CZ	*Nothophytophthora* taxon 2	MO038	OP910307	OP903810	
CZ	*Phy. citrinum*	MO335	OP910308	OP903811	
CZ	*Phy. litorale*	MO018	OP910309	OP903812	

**Country**	**Oomycete species**	**Isolate code**	**ITS**	***coxI***	***ß-tub***
CZ	*Phy.* sp. 1 ROH-2015	MO293	OP910310		
CZ	*Phy.* sp. PV So7	MO219	OP910311	OP903813	
SK	*P. bilorbang*	SK058	OP910295	OP903804	
SK	*P. gonapodyides*	SK008	OP910296	OP903805	
SK	*P. lacustris*	SK003	OP910297	OP903806	
SK	*P. lacustris*	SK162	OP910298	OP903807	
SK	*P. plurivora*	SK154	OP910299	OP903808	
SK	*Nothophytophthora* taxon 1	SK131	OP910312	OP903814	
SK	*Phy. litorale*	SK014	OP910313	OP903815	
SK	*Phy.* sp. 1 ROH-2015	SK089	OP910314	OP903816	
SK	*Py. kashmirense*	SK176	OP910315	OP903817	
SK	*Py.* sp. 1 MNS-2013	SK153	OP910316	OP903818	
SK	*Py.* sp. strain 1-9-like1	SK161	OP910317	OP903819	
SK	*Py.* sp. strain 1-9-like4	SK173	OP910318	OP903820	
