# Supplementary material for: Phytophthora, Nothophytophthora and Halophytophthora diversity in rivers, streams and riparian alder ecosystems of Central Europe
Source: Mycol Prog. 2023 Jun 13;22(7):50. doi: 10.1007/s11557-023-01898-1 (PMC10264269; doi:10.1007/s11557-023-01898-1)
Supplement: Supplementary file 5 — Supplementary file5 (PDF 762 kb) [file 11557_2023_1898_MOESM5_ESM.pdf]

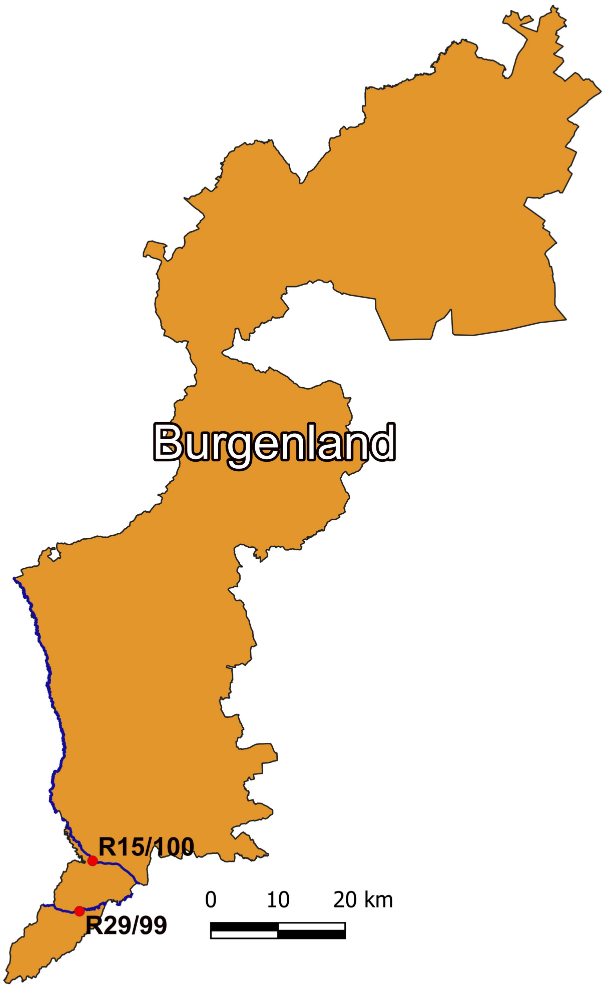

Figure S2. Distribution of sites included in the oomycete survey of watercourses and riparian alder forests in Burgenland (Austria).
