# Supplementary material for: Phytophthora, Nothophytophthora and Halophytophthora diversity in rivers, streams and riparian alder ecosystems of Central Europe
Source: Mycol Prog. 2023 Jun 13;22(7):50. doi: 10.1007/s11557-023-01898-1 (PMC10264269; doi:10.1007/s11557-023-01898-1)
Supplement: Supplementary file 13 — Supplementary file13 (PDF 861 kb) [file 11557_2023_1898_MOESM13_ESM.pdf]

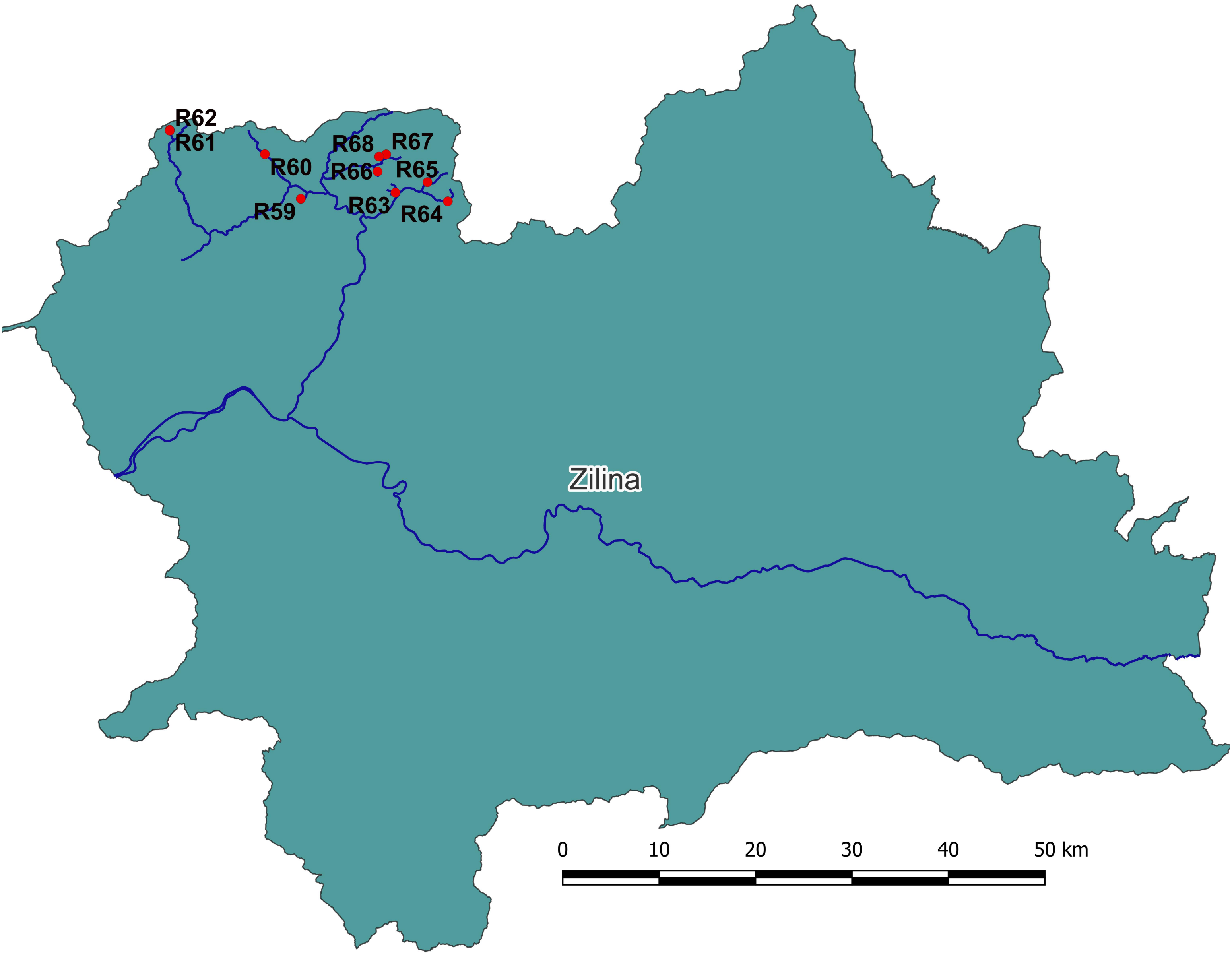

Figure S10. Distribution of sites included in the oomycete survey of forest streams in the Žilina region of Slovakia.
